# FnrL and Three Dnr Regulators Are Used for the Metabolic Adaptation to Low Oxygen Tension in *Dinoroseobacter shibae*

**DOI:** 10.3389/fmicb.2017.00642

**Published:** 2017-04-20

**Authors:** Matthias Ebert, Sebastian Laaß, Andrea Thürmer, Louisa Roselius, Denitsa Eckweiler, Rolf Daniel, Elisabeth Härtig, Dieter Jahn

**Affiliations:** ^1^Institute of Microbiology, Technische Universität BraunschweigBraunschweig, Germany; ^2^Institute for Molecular Biosciences, Goethe-University FrankfurtFrankfurt, Germany; ^3^Göttingen Genomics Laboratory, Institute of Microbiology and Genetics, Georg-August University GöttingenGöttingen, Germany; ^4^Braunschweig Integrated Centre of Systems Biology, Technische Universität BraunschweigBraunschweig, Germany

**Keywords:** anaerobic energy metabolism, *Dinoroseobacter shibae*, regulation, oxygen-dependent gene expression, denitrification, FnrL, Dnr, Crp/Fnr regulator

## Abstract

The heterotrophic marine bacterium *Dinoroseobacter shibae* utilizes aerobic respiration and anaerobic denitrification supplemented with aerobic anoxygenic photosynthesis for energy generation. The aerobic to anaerobic transition is controlled by four Fnr/Crp family regulators in a unique cascade-type regulatory network. FnrL is utilizing an oxygen-sensitive Fe-S cluster for oxygen sensing. Active FnrL is inducing most operons encoding the denitrification machinery and the corresponding heme biosynthesis. Activation of gene expression of the high oxygen affinity *cbb*_3_-type and repression of the low affinity *aa*_3_-type cytochrome c oxidase is mediated by FnrL. Five regulator genes including *dnrE* and *dnrF* are directly controlled by FnrL. Multiple genes of the universal stress protein (USP) and cold shock response are further FnrL targets. DnrD, most likely sensing NO via a heme cofactor, co-induces genes of denitrification, heme biosynthesis, and the regulator genes *dnrE* and *dnrF*. DnrE is controlling genes for a putative Na^+^/H^+^ antiporter, indicating a potential role of a Na^+^ gradient under anaerobic conditions. The formation of the electron donating primary dehydrogenases is coordinated by FnrL and DnrE. Many plasmid encoded genes were DnrE regulated. DnrF is controlling directly two regulator genes including the Fe-S cluster biosynthesis regulator *iscR*, genes of the electron transport chain and the glutathione metabolism. The genes for nitrate reductase and CO dehydrogenase are repressed by DnrD and DnrF. Both regulators in concert with FnrL are inducing the photosynthesis genes. One of the major denitrification operon control regions, the intergenic region between *nirS* and *nosR2*, contains one Fnr/Dnr binding site. Using regulator gene mutant strains, *lacZ-*reporter gene fusions in combination with promoter mutagenesis, the function of the single Fnr/Dnr binding site for FnrL-, DnrD-, and partly DnrF-dependent *nirS* and *nosR2* transcriptional activation was shown. Overall, the unique regulatory network of the marine bacterium *D. shibae* for the transition from aerobic to anaerobic growth composed of four Crp/Fnr family regulators was elucidated.

## Introduction

The heterotrophic Alphaproteobacterium *Dinoroseobacter shibae* DFL12^T^ is a member of the *Roseobacter* group, which are highly abundant in the marine ecosystem and possess a large metabolic diversity (Buchan et al., 2005; Wagner-Döbler and Biebl, 2006; Simon et al., 2017). *D. shibae* DFL12^T^ utilizes carbon sources usually via the Entner-Doudoroff-pathway instead of standard glycolysis (Fürch et al., 2009). The marine bacterium is able to perform aerobic anoxygenic photophosphorylation to gain additional energy (Biebl et al., 2005). Furthermore, anaerobic growth of *D. shibae* DFL12^T^ using nitrate as terminal electron acceptor was proposed (Wagner-Döbler et al., 2010). It was shown, that upon depletion of the electron acceptor oxygen *D. shibae* DFL12^T^ establishes the whole process of denitrification with the reduction of nitrate via nitrite, nitric oxide, nitrous oxide to dinitrogen (Laass et al., 2014). The corresponding denitrification gene cluster comprises 39 genes organized in six operons. A fine-tuned regulatory network was expected for the gene expression control in response to low oxygen tension (Zumft, 1997). Within the denitrification gene cluster two genes, Dshi_3189 and Dshi_3191, encoding members of the Crp/Fnr family of transcription factors were found. Within the whole genome of *D. shibae* a total of seven genes encoding Crp/Fnr-like regulators were identified (Wagner-Döbler et al., 2010).

The superfamily of Crp/Fnr-like transcription factors are known to respond to a broad spectrum of intracellular and exogenous stimuli (Körner et al., 2003). Despite their low amino acid sequence identity of 25% this group of transcription factors shares common structural features. Known Crp/Fnr family proteins usually consist of two functionally distinct domains, a DNA binding helix-turn-helix motif and an N-terminal region of multiple antiparallel β-strands forming the sensory domain (Schultz et al., 1991). The sensing regions are individually adapted for the detection of highly different signals (Green et al., 2001). *Escherichia coli* Crp reversibly binds cAMP to monitor the glucose status of the cell (Crothers and Steitz, 1992). Fnr, the global oxygen-dependent transcriptional regulator for fumarate and nitrate reduction was firstly characterized for *E. coli* (Spiro and Guest, 1990; Khoroshilova et al., 1997). The sensory region of *E. coli* Fnr possesses four conserved cysteine residues (C20, C23, C29, and C122) at its N-terminus that mediate *in vivo* activity via ligation of an oxygen-sensitive iron-sulfur cluster (Trageser and Unden, 1989; Kiley and Reznikoff, 1991; Green and Guest, 1993; Kiley and Beinert, 1998). An intact iron-sulfur cluster is necessary for dimerization of the regulator and subsequent binding to the palindromic Fnr binding site **TTGAT**-N_4_-**ATCAA** (Green et al., 1996; Lazazzera et al., 1996). *E. coli* Fnr in cooperation with the nitrate/nitrite responsive two component regulatory systems NarX/L and NarP/Q and the redox regulator ArcA/B is controlling the complex network of multiple anaerobic primary dehydrogenases, terminal oxidases and mixed acid fermentation processes (Jahn and Jahn, 2012; Tielen et al., 2012). In *Bacillus subtilis* Fnr in combination with the two component redox responsive system ResDE, the nitric oxide sensor NsrR, the Rex regulator responding to changes in the cellular NAD^+^/NADH ratio and the acetate sensor AlsR are regulating the fine-tuned regulatory network of the anaerobic energy metabolism (Härtig and Jahn, 2012). For *Pseudomonas aeruginosa* regulation of denitrification genes was found dependent on Anr, a homolog of Fnr, a second Crp/Fnr-family regulator Dnr, which senses NO and the nitrate responsive NarX/L system (Schreiber et al., 2007; Rinaldo et al., 2012). All three regulators induce the expression of the nitrate reductase genes *narGHJI* (Schreiber et al., 2007). Dnr activates the expression of residual denitrification genes including *nirS, nirQ, norC*, and *nosR* (Arai, 2003). Active Anr binds to a conserved palindromic sequence within the target promoters known as Anr box, which is similar to the Fnr binding site. The Dnr binding site is indistinguishable from the Anr box (Rompf et al., 1998). A fine-tuned interplay of four Crp/Fnr-like regulators, FnrA, DnrD, DnrE, and DnrS with NarX/L is required for the regulating the denitrification genes of *Pseudomonas stutzeri* (Härtig and Zumft, 1999; Vollack et al., 1999; Vollack and Zumft, 2001). In *Rhodobacteraceae* regulators including RegA/B, FnrL, AppA/PpsR, and PrrBA are responsible for the coordination of the anaerobic metabolism (Wu and Bauer, 2008; Winkler et al., 2013; Kumka and Bauer, 2015).

For *D. shibae* time resolved transcriptome and proteome analyses of a continuous culture that was shifted from aerobic to nitrate respiratory conditions revealed the induced expression of four potential Crp/Fnr-like genes Dshi_0660, Dshi_3189, Dshi_3191, and Dshi_3270 (Laass et al., 2014). Due to their potential involvement in the aerobic to anaerobic transition process, they were annotated as FnrL, DnrD, DnrE, and DnrF, respectively. However, only FnrL contains cysteine residues potentially involved in iron-sulfur cluster formation. No genes for other typical redox sensing or nitrate/nitrite responsive regulators were found in the *D. shibae* genome. Here, we elucidated the regulatory network controlled by these four Crp/Fnr-family regulators. Their regulons were defined using regulatory mutants and transcriptome analyses. Promoter activities crucial for the onset of denitrification in *D. shibae* were further investigated *in vivo* using promoter reporter gene fusions. The iron-sulfur cluster of FnrL was demonstrated for the recombinant purified protein. A novel cascade type regulatory scenario for the aerobic-anaerobic transition coordinated by four Crp/Fnr regulators was observed.

## Materials and methods

### Bacterial strains and growth conditions

The type strain *D. shibae* DFL12^T^ and corresponding mutant strains were grown routinely aerobically in Marine-Bouillon (MB, Roth, Karlsruhe, Germany) at 30°C in bottle flasks shaking at 200 rpm in the dark or on the same medium solidified with 1.5% agar. *E. coli* strains were routinely grown at 37°C and shaking at 200 rpm in Lysogenic Broth (LB) supplemented with the appropriate antibiotics and amino acids (Table 1). The growth behavior of the *D. shibae* strains was analyzed under aerobic and anaerobic conditions in artificial seawater medium (SWM; Tomasch et al., 2011) supplemented with 16.9 mM succinate in bottle flasks shaking at 200 rpm for aerobic growth. For anaerobic cultivation NaNO_3_ to the final concentration of 25 mM was added and incubation was performed in serum flasks sealed with rubber stoppers shaking at 100 rpm (Ebert et al., 2013).

**Table 1:** **Strains and plasmids used in this study**.

**Strain**	**Description**	**Sources/References**
***D. shibae*** **STRAINS**		
DFL12^T^	wild type	Biebl et al., 2005
DS001	*ΔfnrL*:: *aacC1* (*Gm*^*r*^)	This study
DS005	*ΔfnrL*:: *aacC1* (*Gm*^*r*^)*;* pRhokS*fnrL, Cm*^*r*^	This study
DS002	*ΔdnrD*:: *aacC1* (*Gm*^*r*^); GFP	This study
DS006	*ΔdnrD*:: *aacC1* (*Gm*^*r*^); GFP; pRhokS*dnrD, Cm*^*r*^	This study
DS003	*ΔdnrE*:: *aacC1* (*Gm*^*r*^)	This study
DS007	*ΔdnrE*:: *aacC1* (*Gm*^*r*^); pRhokS*dnrE, Cm*^*r*^	This study
DS004	*ΔdnrF*:: *aacC1* (*Gm*^*r*^)	This study
DS008	*ΔdnrF*:: *aacC1* (*Gm*^*r*^); pRhokS*dnrF, Cm*^*r*^	This study
DS100	*DFL12^T^; pBBR1nirS-lacZ, Cm*^*r*^	This study
DS101	*ΔfnrL*:: *aacC1* (*Gm*^*r*^); *pBBR1nirS-lacZ, Cm*^*r*^	This study
DS102	*ΔdnrD*:: *aacC1* (*Gm*^*r*^); *pBBR1nirS-lacZ, Cm*^*r*^	This study
DS103	*ΔdnrE*:: *aacC1* (*Gm*^*r*^); *pBBR1nirS-lacZ, Cm*^*r*^	This study
DS104	*ΔdnrF*:: *aacC1* (*Gm*^*r*^); *pBBR1nirS-lacZ, Cm*^*r*^	This study
DS126	*DFL12^T^; pBBR1nirS(mu)-lacZ, Cm*^*r*^	This study
DS127	*ΔfnrL*:: *aacC1* (*Gm*^*r*^); *pBBR1nosR2-lacZ, Cm*^*r*^	This study
DS128	*ΔdnrD*:: *aacC1* (*Gm*^*r*^); *pBBR1nosR2-lacZ, Cm*^*r*^	This study
DS129	*ΔdnrE*:: *aacC1* (*Gm*^*r*^); *pBBR1nosR2-lacZ, Cm*^*r*^	This study
DS130	*ΔdnrF*:: *aacC1* (*Gm*^*r*^); *pBBR1nosR2-lacZ, Cm*^*r*^	This study
***E. coli*** **STRAINS**		
DH10b	*F- endA1 recA1 galE15 galK16 nupG rpsL ΔlacX74 Φ80lacZΔM15 araD139 Δ(ara, leu)7697 mcrA Δ(mrr-hsdRMS-mcrBC) λ-*	Invitrogen
ST18	*E. coli* S17-1*ΔhemA thi pro hsdR*-M- chromosomal integrated [*RP4-2 Tc::Mu:Kmr::Tn7, Tra+ Trir Strr*]	Thoma and Schobert, 2009
Bl21(DE3) pLysS	*F- ompT gal dcm lon hsdSB(rB- mB-) λ(DE3) pLysS(cm^*r*^)*	Stratagene
**PLASMIDS**		
pEX18Tc	Tc^*r*^; oriT^+^, sacB^+^, *lacZ*,	Hoang et al., 1998
pEX18Ap	*Amp*^*r*^; oriT^+^, sacB^+^, *lacZ*,	Hoang et al., 1998
pEX18Gm	*Gm*^*r*^; oriT^+^, sacB^+^, *lacZ*,	Hoang et al., 1998
pBBR1MCS	*Cm*^*r*^; *lacZ* P*_*lac*_* P*_*T*7_ rep*	Kovach et al., 1994
pBBR1MCS-5	*Gm*^*r*^; *lacZ* P*_*lac*_* P*_*T*7_ rep*	Kovach et al., 1995
pPS858	*Amp*^*r*^; *Gm*^*r*^; GFP	Hoang et al., 1998
pEX18Δ*fnrL*	*Tc*^*r*^; *fnrl*:: *aacC1*	This study
pEX18Δ*dnrD*	*Tc*^*r*^; *dnrD*:: *aacC1*	This study
pEX18Δ*dnrE*	*Tc*^*r*^; *dnrE*:: *aacC1*	This study
pEX18Δ*dnrF*	*Amp*^*r*^; *dnrF*:: *aacC1*	This study
pRhokS*fnrL*	*Chl*^*r*^; *fnrL*	This study
pRhokS*dnrD*	*Chl*^*r*^; *dnrD*	This study
pRhokS*dnrE*	*Chl*^*r*^; *dnrE*	This study
pRhokS*dnrF*	*Chl*^*r*^; *dnrF*	This study
pMA-T	*Amp*^*r*^; Col E1 origin	Thermo Fisher
13AADNLP_seq3_dnrD_pMA-T	*Amp*^*r*^; Col E1 origin; *dnrD*	Thermo Fisher
pET52FnrL	*Amp*^*r*^; *fnrL*	This study
pBBRLIC-*lacZ*	*Cm*^*r*^, LIC-*lacZ*	This study
mini-CTX-*lacZ*	*Tc*^*r*^, Col E1 mini CTX vector carrying the *lacZ* fusion	Becher and Schweizer, 2000
pBBR*nirS*-*lacZ*	*Cm*^*r*^, *nirS-lacZ*	This study
pBBR*nirS(mu)*-*lacZ*	*Cm*^*r*^, *nirS(mu)-lacZ*	This study
pBBR*nosR2*-*lacZ*	*Cm*^*r*^, *nosR2-lacZ*	This study

### Construction of vectors for recombinant FnrL production

The *fnrL* gene (Dshi_0660) was PCR amplified using primers oPT229 and oPT230 (Table 2) containing *Sma*I and *Eco53*kI restriction sites from *D. shibae* genomic DNA. The amplification product and the pET52b vector (Novagen, Darmstadt, Germany) were both digested using *Sma*I and *Eco53*kI. Ligation of both DNA fragments resulted in the plasmid pET52FnrL.

**Table 2:** **Primers used in this study**.

**Name**	**Sequence (5′–3′)^a^**
oPT204	TCCGTGTAGCCCTGGAAATT
oPT205	ATGTGCTTTTCCTTGCGGTT
oPT206	TGGCCGGCATTGGATTGATC
oPT207	TCTCAGTTTGTTCCCGTAGCG
oPT208	ATCCAATGCCGGCCAGACGCACACCGTGGAAA
oPT209	GGGAACAAACTGAGAGCGGCGTTGTGACAATTT
oPT222	GAAGTGCCTTCACCTTCGAG
oPT223	CGCACAGACATGCGTACTTT
oPT224	CAATTCGTTCAAGCCGAGAT
oPT239	CAGAGAGCTCATTTCAGCCAGCAGGGTATC
oPT240	TGCACTGCAGGTGACGCTACGGGAACAAAC
oPT241	AGACCGACGAGATGGAATTG
oPT242	GGGGTGGTGTTCATCATCTG
oPT227	GTTCATGCGACGATTAAGCA
oPT228	CTCTACATGGTTGGCTGACG
oPT169	GCGCTGCGGCCCTACTGGAGCTGAATTGGGGATCTTGAAGTTC
oPT170	CGAGCGTCAGCCAACCGAGCTCGAATTAGCTTC
EH645	CAGAGAGCTCGGCGACCCGGTGAAGTGC
EH646	TGCACTGCAGCTCGCGGGCGCTACATCA
oPT235	TGTATCGAATCACCCCCAAT
oPT236	CTTGTCATAGGGCAGCATCA
oPT216	AGCGTGTCCGAGATGAACTT
oPT217	GATCACCATCACCGTCACC
oPT218	CTTGTCCATCCGGGTGAA
oPT219	ATTTGCCGACAACCGTGT
oPT220	CCCGGATGGACAAGGACGCACACCGTGGAAA
oPT221	CGGTTGTCGGCAAATGCGGCGTTGTGACAATTT
DnrEFW	CAGAGAGCTCCTAGGACATCGGGGTGCTG
DnrEBW	TGCACTGCAGTGCGACGAAAAGGCTCAG
oPT176	CCAAGGCTAAAACCGCACT
oPT177	GAACGAACACGGGCTCAC
oPT146	GACGGTACCCTGATGACGGGCGAGAAC
oPT147	GACGAGCTCCATCCTTGATCTGTGCCA
oPT148	GACGAGCTCGGAGAGGCACATGACACCT
oPT149	GACAAGCTTGTAAAGGCCGGTGACGATG
DnrFFW	CAAGAGAGCTCGTGGTGTCCCATCCACAAA
DnrFBW	TGCACTGCAGCCGCGCCAAACTAGCATC
oPT237	AGGGTCTTGTCGGAATGATG
oPT238	TCGGCAAGCTCAGGACTATT
oPT229	TCCCCCGGGATGCCGGCCACCAG
oPT230	TCGAGCTCTCAGACCAGCGGGCC
EH635	GCTCTAGATTTTACCGCGGGCTTTCCCGGGAAGGAGGAACTACTATGACCATGATTACGGATTC
EH636	CCGCTCGAGTTTCCTTACGCGAAATACG
EH675	CCGCGGGCTTTCCCAGCCTCAGGATGTCTCCATTT
EH676	GTTCCTCCTTCCCACCTGCATGGTGCAGAGTAT

a *Restriction enzyme sites are underlined*.

### Production and purification of recombinant FnrL

For production of heterologous FnrL *E. coli* BL21(DE3) pLysS strain carrying the pET52FnrL vector was grown in 500 ml LB-medium containing 100 μg/ml of ampicillin at 37°C and 200 rpm in a 1,000 ml flask. The medium was inoculated to a starting OD_578 nm_ of 0.05 with a corresponding overnight culture. After reaching an OD_578 nm_ of 0.5–0.6, isopropyl-β-D-thiogalactopyranosid (IPTG) was added to a final concentration of 50 μM to induce protein production. The cultures were shifted to 17°C and 100 rpm for 16 h. Next the cultures were shifted to anaerobiosis and incubated for 2 h. All of the following procedures were performed under strict anaerobic conditions. A cell pellet was obtained by centrifugation of the culture for 15 min at 4,000 × g at 4°C. The cell pellet was resuspended in 10 ml binding buffer (100 mM Tris-HCl pH 7.5, 150 mM NaCl). For cell disruption, a French press (1,200 p.s.i.) was used and a soluble protein fraction was obtained by ultracentrifugation (40,000 × g, 65 min, 4°C). The supernatant was loaded onto a 1 ml *Strep*-Tactin® Superflow® high capacity column (IBA GmbH, Goettingen, Germany). The column was washed two times with 10 ml of washing buffer (100 mM Tris-HCl pH 7.5, 150 mM NaCl). The bound proteins were eluted with 10 ml of elution buffer (100 mM Tris-HCl pH 7.5, 150 mM NaCl, 2 mM desthiobiotin). The purified recombinant proteins were stored at 17°C under oxygen exclusion. Protein fractions were analyzed by SDS-polyacrylamide gel electrophoresis (SDS-PAGE; Laemmli, 1970; Righetti, 1990). Protein concentrations were determined using the Bradford Reagent (Sigma-Aldrich, St. Louis, USA) according to the manufacturer‘s instructions.

### Reconstitution of iron-sulfur clusters in FnrL

The chemical reconstitution of iron-sulfur clusters in FnrL was performed with a final concentration of 40 μM protein solution under strictly anaerobic conditions in a Coy anaerobic chamber (Coy, Grass Lake, USA). Initially, 10 mM DTT were added to the protein solution and incubated for 1 h at 17°C. Afterwards, 1 mM ammonium iron citrate was slowly added under careful mixing. After 5 min of incubation 1 mM lithium sulfide was added. The reaction was stopped after reaching a brownish color (~15 min) by 5 min centrifugation at 14,000 × g. The reconstituted protein was purified using a NAP column (GE Healthcare, Solingen, Germany). Spectroscopic analysis was performed using a UV/Vis Spectroscope V-550 (Jasco, Gross-Umstadt, Germany).

### Construction of *D. shibae* deletion mutants

To obtain deletion strains of *fnrL* (Dshi_0660), *dnrD* (Dshi_3189), *dnrE* (Dshi_3191), and *dnrF* (Dshi_3270) three different cloning strategies were used. For deletion of *dnrD* a synthetic insert was designed and synthesized by GeneArt (Thermo Fisher Scientific, Waltham, USA). The insert harbored 500 bp upstream sequences of *dnrD* and 500 bp downstream sequences. The *dnrD* gene was replaced on the insert by the gentamicin resistance cassette of plasmid pEX18Gm (Hoang et al., 1998). This synthetic insert was cloned by flanking *Kpn*I and *Hind*III restriction sites into the equally cutted pMA-T vector (GeneArt, Thermo Fisher Scientific, Waltham, USA) resulting in vector 13AADNLP_seq3_dnrD_pMA-T (GeneArt, Thermo Fisher Scientific, Waltham, USA). Due to a lack in functionality of the ordered gentamicin resistance cassette, the gene was excised again by *Sac*I cleavage and replaced by the *aacC1* gene (*Gm*^*r*^) and the *gfp* gene from vector pPS858 (Hoang et al., 1998). The newly created insert was cleaved by *Kpn*I and *Hind*III and ligated into the suicide vector pEX18Tc resulting in the final pEX18Δ*dnrD* vector. For construction of the *fnrL* mutant strain the CloneEZ® PCR cloning Kit (Genscipt, New Jersey, USA) was used. A 2715 bp fragment containing the *fnrL* gene together with 993 bp upstream and 973 bp downstream sequences was amplified from genomic DNA of *D. shibae* using primers oPT204 and oPT205 (Table 2). This PCR fragment was cloned into a pEX18Tc vector cleaved by *Eco53*kI by blunt end ligation. The primers oPT206 and oPT207 were used to amplify the entire vector, lacking the full length open reading frame of *fnrL*. Next, the gentamicin resistance cassette from plasmid pBBR1MCS-5 (Kovach et al., 1995) was PCR amplified using primers oPT208 and oPT209. The obtained insert and vector backbone was assembled by using the CloneEZ PCR Cloning Kit (GenScript, Piscataway, USA) according to the manufacturer's instructions resulting in pEX18Δ*fnrL*. The construction of the *dnrE* knockout vector was performed by using the same experimental procedure and resulted in vector pEX18Δ*dnrE*. Using primers oPT216 and oPT217 the full length *dnrE* gene together with 1,178 bp upstream and 1,136 bp downstream sequences of *dnrE* were amplified. For vector amplification primers oPT218 and oPT219 were used. The gentamicin resistance cassette was amplified from pBBR1MCS-5 (Kovach et al., 1995) using oPT220 and oPT221. To obtain a *dnrF* mutant strain of *D. shibae* a *Sac*I digested *aacC1* (*gm*^*r*^) gene of pPS858 was ligated between two PCR fragments of the upstream and downstream region of *dnrF*. Using primers oPT146, which contained a *Kpn*I restriction site at the 5′ end and oPT147 with a *Sac*I restriction site, a 686 bp upstream fragment was obtained. The 541 bp downstream fragment of *dnrF* was amplified using oPT148 with a *Sac*I restriction site and oPT149 which contained a *Hind*III restriction site. The resulting insert was cloned into a pEX18Ap vector (Hoang et al., 1998) resulting in vector pEX18Δ*dnrF*. The obtained suicide vectors for *fnrL, dnrD, dnrE*, and *dnrF* were transferred into *D. shibae* DFL12^T^ via biparental mating using the *E. coli* ST18 donor strain as described previously (Thoma and Schobert, 2009; Ebert et al., 2013). The gene knockout was obtained by double-homologous recombination and was selected on half-concentrated marine agar plates containing 80 μg/ml gentamicin. The genomic structure of the mutant strains DS001(Δ*fnrL*), DS002(Δ*dnrD*), DS003(Δ*dnrE*), and DS004(Δ*dnrF*) constructed in this study were confirmed by PCR (Table 2).

For complementation, the *fnrL* gene together with 324 bp upstream sequences was PCR amplified using genomic DNA of *D. shibae* and the primers oPT239 and oPT240 containing a *Sac*I and a *Pst*I restriction site, respectively. The insert was ligated into the multiple cloning site of a *Sac*I and *Pst*I digested pRhokS vector (Katzke et al., 2010), resulting in vector pRhokS*fnrL*. The same procedure was used for construction of complementation vectors of *dnrD, dnrE*, and *dnrF*. For construction of the *dnrD* expression plasmid primers EH645 and EH646 were used to amplify a 1,022 bp DNA fragment containing the *dnrD* gene and 301 bp upstream sequences resulting in vector pRhokS*dnrD*. For the *dnrE* vector primers DnrEFW and DnrEBW were used to amplify a 1,050 bp DNA fragment which contained the *dnrE* gene together with 334 bp upstream sequences resulting in vector pRhokS*dnrE*. For the *dnrF* plasmid primers DnrFFW and DnrFBW were used to amplify a 995 bp insert containing the *dnrF* gene together with 232 bp upstream sequences resulting in vector pRhokS*dnrF*. Complementation vectors were transferred into the respective mutant strains via biparental mating using the *E. coli* ST18 donor strain. Selection was performed on half-concentrated marine agar plates containing 15 μg/ml chloramphenicol. The resulting complemented *D. shibae* strains were termed DS005(Δ*fnrL*, pRhokS*fnrL*), DS006(Δ*dnrD*, pRhokS*dnrD*), DS007(Δ*dnrE*, pRhokS*dnrE*), and DS008(Δ*dnrF*, pRhokS*dnrF*; Table 1).

### Growth curve and shift experiments

To record growth curves a pre-culture of the *D. shibae* strain of interest was inoculated in SWM and grown overnight at 30°C and 200 rpm in dark. Then 125 ml main culture was inoculated to a final OD_578nm_ of 0.05 in a 1,000 ml baffled flask. For anaerobic growth the culture was inoculated to a final OD_578 nm_ of 0.3 in a serum flask sealed with a rubber stopper. For shift experiments the main culture was inoculated to a final OD_578 nm_ of 0.05 in SWM in a shaking flask. After reaching on OD_578 nm_ of ~0.5 the cultures were shifted to anaerobic conditions and 25 mM (f.c.) NaNO_3_ was supplied. For anaerobic cultivation a serum flask was used. Oxygen tension was measured every 5 min using a PreSense Fibox 3 LCD trace v7 and an oxygen sensor Type PSt3 with an accuracy of ±0.15%. Samples were taken for RNA preparation directly before the shift, and after 30 and 60 min of anaerobic conditions.

### RNA isolation

For RNA isolation a volume of 2 ml cell culture was removed from the culture and transferred in 4 ml of RNAprotect (Qiagen, Hilden, Germany). The solution was roughly mixed and incubated for 5 min at room temperature. Cells were collected by centrifugation at 4,000 × g for 10 min at 4°C, washed with 1 ml water and centrifuged at 4,000 × g for 5 min. The resulting cell pellet was frozen and stored at −80°C until further processing. Cells were enzymatically lysed (15 mg/ml lysozyme) and mechanically disrupted by using 70–150 μm glass beads and a vortex genie 2 (Scientific industries, Inc., New York, USA) for 30 min. Samples were centrifuged at 4,000 × g for 3 min at 4°C and the supernatant was transferred to fresh tube followed by the addition of 1:3 (v/v) 100% EtOH. Samples were loaded onto a RNeasy Mini Spin column (RNeasy mini kit, Qiagen, Hilden, Germany). Genomic DNA was removed using an on column DNase I treatments for 15 min. Residual DNase I was removed by following the RNeasy mini kit manufacture guidelines. The eluted RNA fraction was treated a second time with DNase I for 15 min and purified by an additional RNeasy Mini Spin column according to the manufacture protocol (RNeasy mini kit, Qiagen, Hilden, Germany).

### RNA microarray

Two micrograms of total RNA was labeled with Cy3 using the ULS-system (Kreatech, Amsterdam, Netherlands) according to the manufacturer's manual. The labeled RNA (600 ng) was fragmented and hybridized to the custom designed gene specific DNA microarray (Agilent, Santa Clara, USA 8 × 15K format) according to Agilent's one-color microarray protocol. Microarray analyses were performed with three technical and three biological replicates. Only genes with a logarithmic change of ≥0.8 when comparing aerobic (0 min) and anaerobic (60 min) expression levels of wild type and mutant strains with a *P* < 0.05 were considered in subsequent analyses. Generated data have been deposited in NCBI's Gene Expression Omnibus (Edgar, 2002) and are accessible through Geo Series accession number GSE93652.

### RNA sequencing

RNA sequencing was performed by the Goettingen genomics laboratory (G2L). Library construction started with total *D. shibae* RNA. The rRNA was depleted with the Ribo-Zero rRNA Removal Kit (Illumina, San Diego, USA). Strand-directed RNA-seq libraries were generated using NEBNext® Ultra™ Directional RNA Library Prep Kit for Illumina® (New England Biolabs). Sequencing was done with MiSeq Reagent Kit v3 (150 cycle) chemistry in a 2 × 75 bp PE-run using the MiSeq Instrument (Illumina, San Diego, USA).

### Promoter-*lacZ* reporter gene fusions

We developed the vector pBBRLIC-*lacZ* to generate *lacZ* transcriptional fusions via ligation-independent cloning (LIC). Primers EH635 (containing the LIC sequence) and EH636 were used to amplify a 3,168 bp DNA fragment from the plasmid mini-CTX-*lacZ* harboring the *lacZ* gene (Becher and Schweizer, 2000). By this approach the *lacZ* gene was fused with the LIC sequence 5′-TTTTACCGCGGGCTTT**CCCGGG**AAGGAGGAACT-3′ (Botella et al., 2010) containing a central *Sma*I restriction site (bold letters) and a ribosomal binding site (underlined). The amplification product and plasmid pBBR1MCS (Kovach et al., 1994) were both digested with *Xho*I and *Xba*I. Ligation of both DNA fragments resulted in plasmid pBBRLIC-*lacZ*. Promoter fragments suitable for ligation-independent cloning (LIC) were generated by amplification of chromosomal DNA fragments using primers with a 5′-CCGCGGGCTTTCCCAGC-3′ LIC tail sequence added to the forward primer and a 5′-GTTCCTCCTTCCCACC-3′ LIC tail sequence added to the revers primer. The pBBRLIC-*lacZ* plasmid was linearized using *Sma*I, gel-purified and treated with T4 DNA polymerase in the presence of 2.5 mM dATP for 20 min at 22°C, followed by 30 min at 75°C to inactivate the enzyme. This generated 12 nt single-stranded overhangs on either side of the *Sma*I restriction site. For ligation independent cloning 0.2 pmol of each amplified promoter fragment was incubated with 2.5 mM dTTP and T4 DNA polymerase. Promoter fragments were then treated with T4 polymerase in the presence of dTTP for 20 min at 22°C followed by enzyme inactivation for 30 min at 75°C. This approach generated inserts with single-stranded ends that are complementary to those of the treated vector. Treated plasmids (5 ng) and inserts (15 ng) were mixed, annealed at room temperature for 10 min and transformed into *E. coli*. The promoter region of *nosR2* was amplified using primers pair EH675 and EH676 and cloned as described above into pBBRLIC-*lacZ* resulting in the pBBR*nosR2*-*lacZ* plasmid. A 118 bp *nirS* DNA fragment corresponding to promoter sequences from position −88 to +30 with respect to the transcriptional start site of *nirS* together with the LIC tail sequences was ordered as synthetic double stranded GeneArt string (Thermo Fisher Scientific Inc., Waltham, USA) and cloned into pBBRLIC-*lacZ* resulting in plasmid pBBR*nirS*-*lacZ*. For *nirS(mu)* the DNA fragment as described for *nirS* was used, however, carrying base exchanges at position −48/−47 from TT to GC and at position −36/−35 from AA to GC. It was ordered as synthetic double stranded GeneArt string (Thermo Fisher Scientific Inc., Waltham, USA). Cloning into pBBRLIC-*lacZ* resulted in plasmid pBBR*nirS(mu)*-*lacZ*.

### ß-galactosidase assay

For ß-galactosidase assays, *D. shibae* cells were grown in salt water medium and harvested after 16 h in the mid-exponential growth phase. ß-Galactosidase assays were performed as described previously (Miller, 1992; Härtig et al., 2004).

## Results

### Classification of Fnr- and Dnr-type regulators of *D. shibae*

Previous investigations identified two different major life styles for the marine bacterium *D. shibae*. Under aerobic growth conditions oxygen-dependent respiration is combined with anoxygenic photosynthesis, while anaerobic growth utilizes denitrification as major path of energy generation (Laass et al., 2014). In many other bacteria Fnr- and Dnr-type regulators were found to coordinate the transition between these two life styles at the transcriptional level (Härtig and Jahn, 2012; Tielen et al., 2012). Initial database searches using the amino acid sequences of *Rhodobacter capsulatus* FnrL and *Pseudomonas aeruginosa* Dnr identified seven open reading frames (Dshi_0660, Dshi_0447, Dshi_2521, Dshi_2528, Dshi_3189, Dshi_3191, Dshi_3270) encoding potential Crp/Fnr-family regulators.

**Figure 1:**
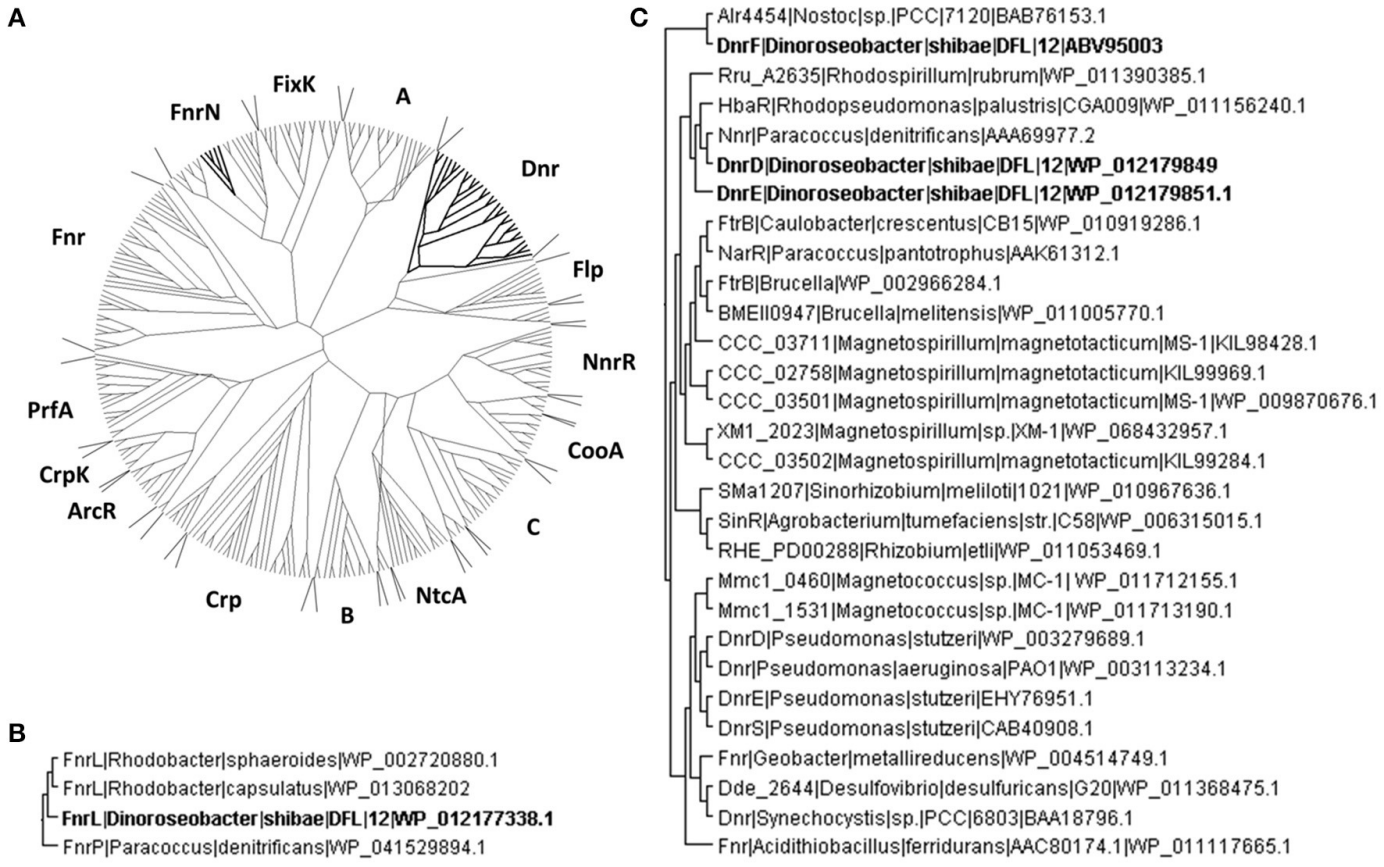
**Phylogenetic affiliations of the D. shibae Crp/Fnr-like regulators. (A)** The unrooted neighbor joining trees containing *D. shibae* Crp/Fnr like regulators affiliated to the Crp/Fnr superfamily are shown. **(B)** FnrN subfamily of Fnr like regulators containing FnrL of *D. shibae*. **(C)** Dnr subgroup of Crp/Fnr like regulators containing three different Dnr regulators of *D. shibae*. The members of the tree are identified by the name of the host bacterium and the NCBI sequence identification number (accession number).

In order to group these Crp/Fnr regulators according to the classification developed by Körner et al. (2003) comprehensive amino acid sequence alignments were performed by using the multiple sequence alignment tool T-coffee (Notredame et al., 2000). A dataset of 287 pair-wise aligned sequences was generated (Figure 1A). The protein encoded by Dshi_0660 affiliated with the FnrN subgroup (Figure 1B). The highest score of 70.16% identity was found for the transcriptional activator FnrL of *R. capsulatus* (WP_013068202). Consequently, the protein was named *D. shibae* FnrL. Moreover, a sequence identity of 64.66% was obtained for FnrP of *Paracoccus denitrificans* (WP_041529894.1). The predicted transcription regulator encoded by Dshi_0447 was named DnrA. It was found affiliated with the A subgroup of Crp/Fnr regulators with 25% overall amino acid sequence identity (Figure 1A). However, no experimental evidence for the regulation of the metabolic adaptation to anaerobiosis was obtained during this investigation. Similarly, the postulated Crp/Fnr family regulators encoded by Dshi_2521 and Dshi_2528, termed DnrB and DnrC respectively, also do not seem to be involved in the aerobic-anaerobic regulatory network. They belong to the subgroup B of Crp/Fnr regulators (Figure 1A). Moreover, functional predictions for the regulators of interest can sometimes be derived from the analysis of the genomic context (Vollack et al., 1999). However, corresponding analyses of the genomic context did not provide further information for the possible functions of DnrA, DnrB, and DnrC. All three of the corresponding genes are surrounded by genes coding for hypothetical proteins in the genome of *D. shibae*.

The DnrD protein encoded by Dshi_3189 showed an amino acid sequence identity of 45.95% with Nnr of *P. denitrificans* (AAA69977.2), but only 27.48% identity with the amino acid sequence of Dnr from *P. aeruginosa*. DnrD and the protein encoded by Dshi_3191 termed DnrE share 42.29% amino acid sequence identity and affiliated within the Dnr subgroup of the Crp/Fnr family (Figure 1C). The genes encoding DnrD and DnrE are localized within the denitrification gene cluster of *D. shibae*. This indicates a possible role in the regulation of denitrification as shown for Dnr from *P. aeruginosa* (Arai et al., 1995; Schreiber et al., 2007), *P. denitrificans* (Van Spanning et al., 1995), *R. sphaeroides* (Tosques et al., 1996), and *P. stutzeri* (Zumft, 1997; Vollack et al., 1999). A third Dnr-like regulator is encoded by Dshi_3270 and was named DnrF. DnrF shared only 19.64% identity with Dnr of *P. aeruginosa* but 27.14% to transcriptional regulator *alr4454* of *Nostoc* sp. PCC7120 (BAB76153.1). With an overall sequence identity of 21.47% compared to the entire Dnr subfamily DnrF was placed at a basal branch point of the Dnr subfamily of the Crp/Fnr family tree (Figure 1C). It might represent a possible new subfamily of regulators within the Crp/Fnr family. In contrast to the relatively low overall amino acid sequence homology of DnrD, DnrE, and DnrF of *D. shibae* to other Dnr regulators, they show a significant amino acid sequence conservation within their DNA-binding domains and therefore may recognize similar DNA target sequences (Dufour et al., 2010). However, the function of a predicted upstream DNA target sequence has to be proven by transcriptome analyses.

### *D. shibae* FnrL contains an oxygen-sensitive Fe-S cluster

**Figure 2:**
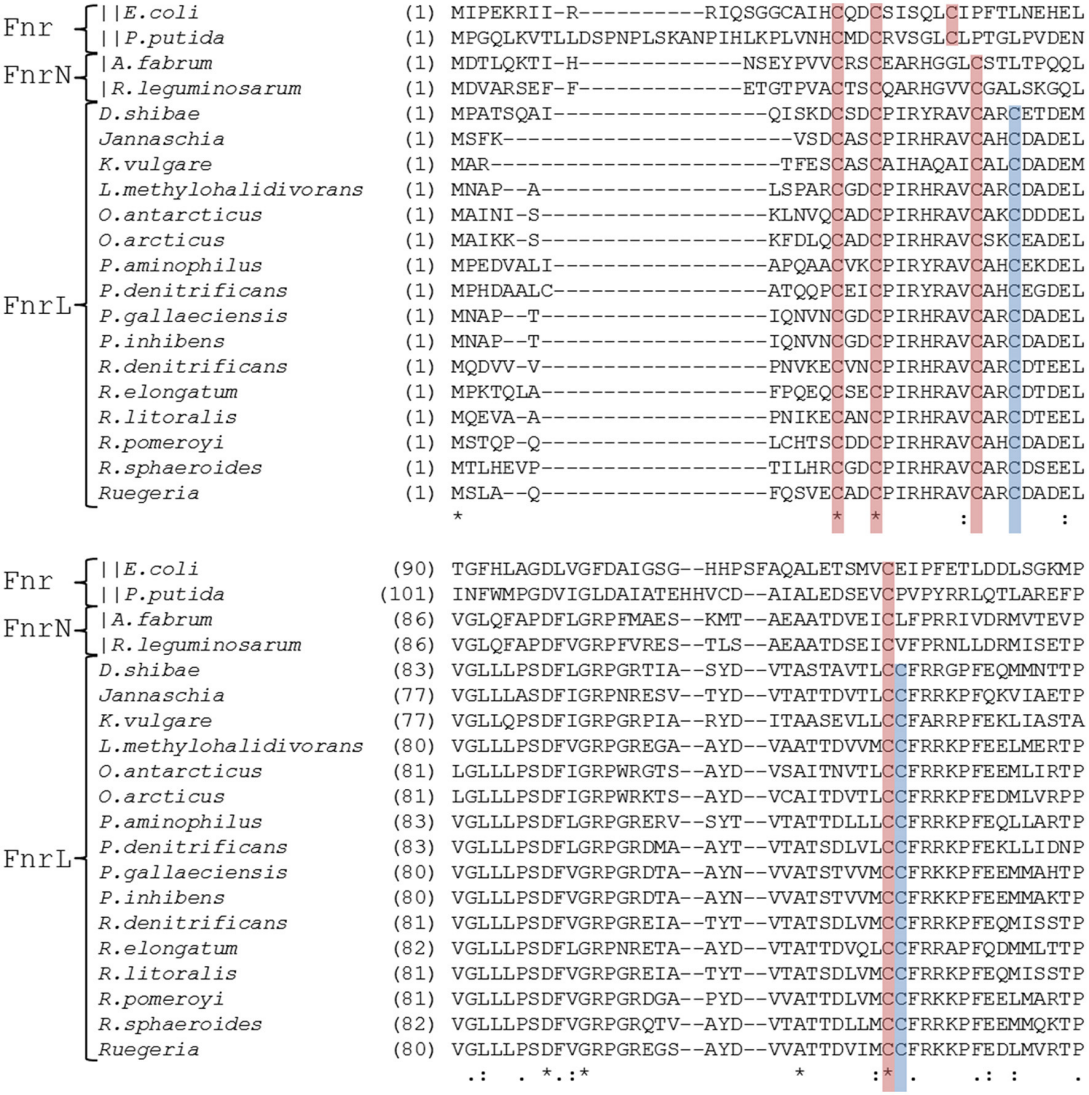
**Alignment of the N-terminal regulatory domains of FnrL like regulators**. Cysteine residues which were known to be essential for activity in *P. denitrificans* are marked in red (Hutchings et al., 2002). Those cysteine residues which were additionally found conserved exclusively in the *Roseobacter* group are indicated in blue. The alignment comprises the amino acid sequences of FnrL proteins from *Dinoroseobacter shibae* DFL12^T^ (WP_012177338.1), *Paracoccus denitrificans* (WP_041529894.1), *Rhodobacter sphaeroides* (WP_002720880.1), *Roseobacter litoralis* (WP_044025718.1), *Roseobacter denitrificans* (WP_044032974.1), *Pheobacter gallaeciensis* (WP_014881157.1), *Phaeobacter. inhibens* (WP_040174583.1), *Roseibacterium elongatum* (WP_025310970.1), *Ruegeria pomeroyi* (WP_011049211.1), *Leisingera methylohalidivorans* (WP_024088845.1), *Ruegeria* sp. (WP_039983362.1), *Jannaschia* sp. (WP_011456972.1), *Paracoccus aminophilus* (WP_020949853.1), *Octadecabacter antarcticus* (WP_015498258.1), *Octadecabacter arcticus* 238 (WP_015493613.1), *Ketoglonicigernium vulgare* (WP_013383280.1). In addition, Fnr from *Escherichia coli* (WP_000611911.1) and Anr from *Pseudomonas putida* (WP_010954166.1) were included. Symbols indicates an entirely conserved column (^*^), a column comprising amino acids of same size and hydropathy (:) and a column comprising amino acids of similar size or evolutionary preserved hydropathy (.) (Notredame et al., 2000).

**Figure 3:**
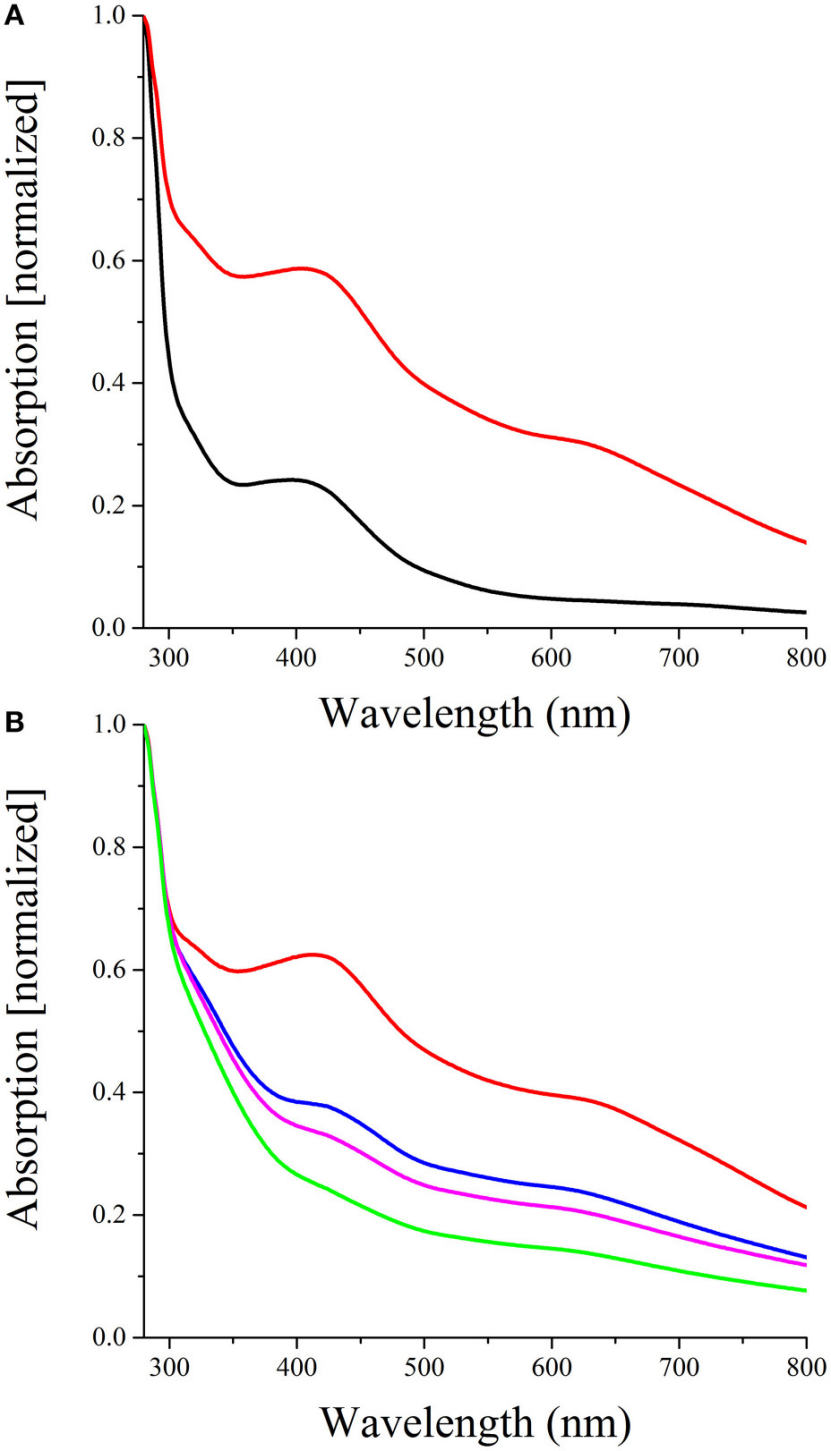
**FnrL contains an oxygen labile Fe-S cluster (A)** Reconstitution of the *D. shibae* DFL12^T^ FnrL Fe-S clusters. The spectrum of 40 μM FnrL (black line) and 20 μM Fe-S cluster reconstituted FnrL (red line) are shown. Samples were normalized by setting absorption at 280 nm to 1. **(B)** Oxygen lability of the FnrL Fe-S cluster. The exposure of Fe-S cluster reconstituted FnrL to air resulted in a decrease of absorption at 420 nm over time [0 min (red line), 60 min (blue line), 120 min (magenta line), and 240 min (green line)].

The two typical features of oxygen sensing Fnr proteins are a highly conserved HTH-motif for promoter recognition and an oxygen-sensitive [4Fe-4S]^2+^ cluster for signal perception. A sequence alignment of *D. shibae* FnrL with homologous proteins of other *Rhodobacteraceae* species identified six highly conserved cysteine residues (Figure 2). For *P. denitrificans* a mutational analysis identified four of these cysteines as functionally relevant for *in vivo* transcriptional activity, most likely as ligands for an Fe-S cluster (Hutchings et al., 2002). In order to investigate *D. shibae* FnrL for the presence of a Fe-S cluster, the regulator protein was produced as a Strep-tagged fusion protein and anaerobically purified to apparent homogeneity. UV/VIS spectroscopy performed with the purified protein revealed a broad shoulder at 420 nm, a typical spectrum for Fe-S cluster containing proteins (Figure 3A). After further reconstitution of Fe-S clusters, an increased absorption at 420 nm was detected. Fe-S cluster integrity upon exposure to air was examined in a time resolved manner using UV/VIS spectroscopy. The absorption at 420 nm at time points of 60, 120, and 240 min were shown in Figure 3B. The Fe-S cluster of *D. shibae* FnrL is obviously oxygen labile since absorption at 420 nm decreased over time. Thus, FnrL is an oxygen-dependent transcriptional regulator of *D. shibae*.

### *D. shibae* knockout mutants of *fnrl, dnrD, dnrE*, and *dnrF* show oxygen tension-dependent growth phenotypes

**Figure 4:**
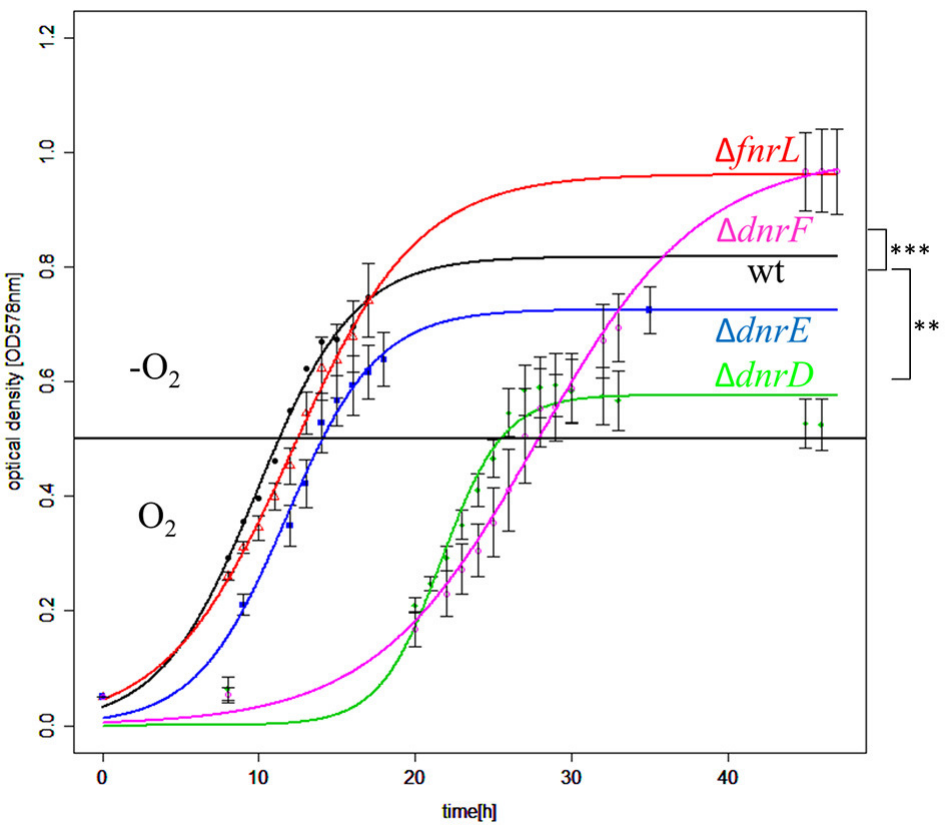
**Growth behavior of ***D. shibae*** Dfl12^**T**^ and oxygen regulatory mutants during an aerobic to anaerobic shift**. *D. shibae* DFL12^T^ wildtype strain (black line), DS001(Δ*fnrL*) (red line), DS002(Δ*dnrD*) (green line), DS003(Δ*dnrE*) (blue line) and DS004(Δ*dnrF*) (magenta line) mutant strains were grown under aerobic conditions in artificial see water medium supplemented with 16.9 mM succinate. After reaching of OD_578 nm_ of 0.5 cells were shifted to anaerobic growth conditions (black horizontal line) and 25 mM sodium nitrate was added. The optical density was measured in three independent replicates and error bars represent the standard deviation. *P*-values were determined by using ANOVA without a *Post-hoc*-test using the wild type values as reference. The symbols indicate *P*-values smaller or equal to 0 (^***^) or 0.001 (^**^).

In order to determine the contribution of FnrL, DnrD, DnrE, and DnrF to aerobic and anaerobic growth, single gene knockout mutant strains were constructed by replacing the encoding genes with gentamicin resistance cassettes. The respective mutant strains DS001(Δ*fnrL*), DS002(Δ*dnrD*), DS003(Δ*dnrE*), DS004(Δ*dnrF*), and the *D. shibae* DFL12^T^ wild type strain were cultivated under aerobic and anaerobic growth conditions and their growth rates were determined (Table 3). The *fnrL* mutant strain DS001 (Δ*fnrL*) grew under aerobic and under anaerobic growth conditions comparable to the wild type strain (Table 3). In contrast, all *dnr* mutant strains DS002(Δ*dnrD*), DS003(Δ*dnrE*), and DS004(Δ*dnrF*) showed reduced growth rates of 0.18, 0.3, and 0.25 per hour compared to the wild type strain with 0.42 per hour under aerobic conditions. Strongly retarded growth was observed for DS002 (Δ*dnrD*) and DS004 (Δ*dnrF*) under anaerobic growth conditions with growth rates of 0.02 and 0.03 per hour compared to the wild type with 0.1 per hour. Complementation of the mutant strains with their intact gene *in trans* (DS005, DS006, DS007, and DS008) resulted in restored aerobic and anaerobic growth to wild type levels for almost all mutant strains. Growth of the complemented Δ*dnrF* strain DS008 under anaerobic conditions was fully restored compared to the wild type strain. In order to test for the contribution of the various regulators to the control of the aerobic to anaerobic transition, we performed shift experiments from aerobic culture in mid-exponential growth phase to anaerobic growth conditions in the presence of nitrate. Growth curves of DS001(Δ*fnrL*), DS002(Δ*dnrD*), DS003(Δ*dnrE*), and DS004(Δ*dnrF*) mutant strains compared to the *D. shibae* DFL12^T^ wild type strain were recorded and the oxygen consumption was recorded until the oxygen was no longer detectable. This was reached 15 min after the shift for each growth curve (Figure 4). Under anaerobic growth condition DS001(Δ*fnrL*) showed almost the same progression like the wild type strain but finally grew to higher cell density before entering the stationary growth phase (Figure 4). Thus, DS001(Δ*fnrL*) might have a growth advantage compared to the wild type under anaerobic conditions. DS003(Δ*dnrE*) showed a slightly reduced growth compared to wild type resulting in a reduced cell number (Figure 4). Strain DS002(Δ*dnrD*) first showed a prolonged lag phase under aerobic conditions but in the exponential growth phase the growth rate was comparable to the wild type. After the shift to anaerobiosis DS002(Δ*dnrD*) stopped growing indicating a clear anaerobic phenotype (Figure 4). Strain DS004(Δ*dnrF*) also showed a prolonged lag phase but after the shift to anaerobic growth conditions, growth increased and the strain finally reached cell numbers higher than the wild type comparable to DS001(Δ*fnrL*). In contrast to the *dnrD* mutant strain DS002(Δ*dnrD*), DS001(Δ*fnrL*), DS003(Δ*dnrE*), DS004(Δ*dnrF*) mutant strains were able to adapt to anaerobiosis and continued growth (Figure 4).

**Table 3:** **Growth rate of ***D. shibae*** DFL12^**T**^ wild type strain and corresponding mutant strains under aerobic and anaerobic growth conditions**.

**Strain**	**Growth rate aerob ± *p* (h^−1^)**	***P*-value aerob**	**Growth rate anaerob± s.d. (h^−1^)**	***P*-value anaerob**
DFL12^T^	0.42 ± 0.02		0.1 ± 0.01	
DS001(Δ*fnrL*)	0.45 ± 0.02	0.5	0.11 ± 0	1
DS005(Δ*fnrL+fnrL*)	0.45 ± 0.03	0.2	0.11 ± 0	1
DS002(Δ*dnrD*)	0.18 ± 0	0	0.02 ± 0	0
DS006(Δ*dnrD+dnrD*)	0.39 ± 0	0.8	0.09 ± 0	1
DS003(ΔdnrE)	0.3 ± 0.04	0	0.1 ± 0	1
DS007(Δ*dnrE*+*dnrE*)	0.39 ± 0.02	0.8	0.1 ± 0	1
DS004(Δ*dnrF*)	0.25 ± 0.01	0	0.03 ± 0.01	0
DS008(Δ*dnrF*+*dnrF*)	0.24 ± 0.01	0	0.08 ± 0.01	0.9

*The growth rates values were determined in the logarithmic growth phase and represent the means of three technical and thee biological replicates with appropriate standard deviation. P-values were determined by using regression and ANOVA followed by a Post-hoc–test with the wild type data as reference. A significant change in growth was assumed for P ≤ 0.05*.

### Definition of the FnrL, DnrD, DnrE, and DnrF regulons

In order to define the different regulons of the four regulators for the elucidation of their contribution to the regulatory network for the anaerobic adaptation, transcriptome analyses were performed. First, the complete aerobic to anaerobic modulon for wild type *D. shibae* was investigated using RNA sequencing. Moreover, all involved open reading frames and corresponding transcriptional start sites became visible. The various mutant strains were analyzed using the DNA array technology. For this purpose shift experiments as outlined above were performed. RNA samples were collected directly before the shift representing the aerobic expression and 60 min after the shift. A differential gene expression was assumed for genes with an absolute log2-fold change higher or equal to 0.8 comparing wild type and mutant strains DS001(Δ*fnrL*), DS002(Δ*dnrD*), DS003(Δ*dnrE*), DS004(Δ*dnrF*). Under anaerobic conditions 329 genes were found induced in the DS001(Δ*fnrL*) mutant strain compared to wild type indicating a major repressor function of FnrL. Concurrently, 160 genes were found repressed, indicating an activating function of FnrL (Table 4). For DS002(Δ*dnrD*) in total 348 genes were found induced indicating a repression through DnrD and 138 were found repressed in a *dnrD* mutational background. Similar counts were found for DS004(Δ*dnrF*) with 316 induced and 92 repressed genes under *dnrF* depletion. Only for DS003(Δ*dnrE*) the regulon seemed to contain less genes. In total 144 genes were found induced and 69 were found repressed within the mutant strain.

**Table 4:** **Comparative analysis of transcriptome results from ***fnrL*** and various ***dnr*** genes mutant strains under anaerobic conditions**.

	**DS001(*ΔfnrL*)**	**DS002 (*ΔdnrD*)**	**DS004 (*ΔdnrF*)**	**DS003 (*ΔdnrE*)**
Activation	160	138	92	69
Repression	329	348	316	144

*Number of differentially expressed genes (alteration in log 2-fold change ≥0.8) within the indicated knockout mutant strains compared to the wild type of D. shibae DFL12^T^*.

### Identification of potential Fnr and Dnr binding sites in the genome of *D. shibae*

By using the Virtual Footprint tool of the PRODORIC database (Münch et al., 2005), for a global genome search of possible Anr, Fnr, and Dnr binding sites, 314 potential Fnr/Dnr binding sites were identified within the genome of *D. shibae*. We correlated these results with differential gene expression data derived from the DNA array analyses using wild type and the regulatory mutant strains. RNA sequencing analyses determined the corresponding transcriptional start sites. The localization of the identified binding sites were given with respect to the transcriptional start site of the regulated genes. Since, we got only a low coverage of transcript by RNA sequencing for the plasmid encoded genes, distances of the identified binding sites were given with respect to the translational start points. These analyses correlated 69 FnrL binding sites with induction or repression of the cognate genes by FnrL (Table S1). For DnrD 10 functional bindings sites, for DnrE 4 binding sites and for DnrF 8 binding sites were identified (Table S2).

### Interplay of FnrL, DnrD, DnrE, and DnrF for the regulation of the denitrification gene cluster

**Figure 5:**
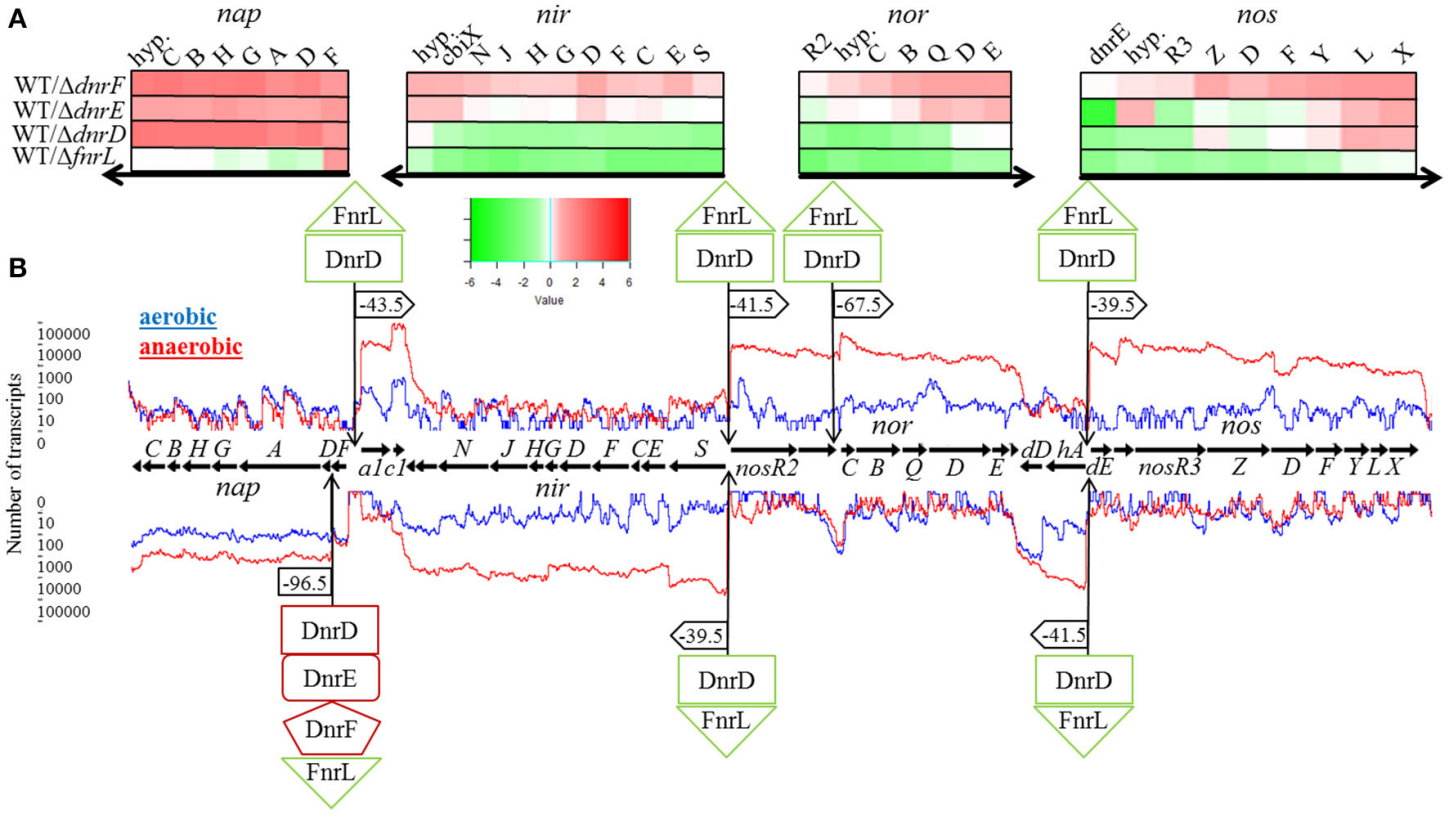
**Regulation of the denitrification operons by FnrL, DnrD, DnrE, and DnrF. (A)** Heat map representation of denitrification gene expression patterns of mutant strains DS001(Δ*fnrL*), DS002(Δ*dnrD*), DS003(Δ*dnrE*), and DS004(Δ*dnrF*) compared to *D. shibae* DFL12^T^ wild type strain grown under anaerobic conditions. The colored bars represent the expression level in log2 scale. Green indicates a relatively low expression level in the mutant strain which indicates activation by the regulator; red indicates relatively high expression levels in the mutant strain compared to the wild type indicating repression. **(B)** Denitrification operons with corresponding RNA sequencing data based transcriptional start sites and transcript quantification under aerobic (blue line) and anaerobic conditions (red line) of the wild type *D. shibae* DFL12^T^. Black horizontal arrows indicate open reading frames and the direction of transcription. Additionally, binding sites of FnrL, DnrD, DnrE, and DnrF are indicated by flags. If the corresponding binding site was located within a promoter sequence, the corresponding distance to the transcriptional start was given. Green boxes indicate an activation by the given regulator, red boxes indicate a repression. *a1, apbE1*; *c1, cycA1*; *hyp*, hypothetical gene; *dD, dnrD*; *hA, hemA3*; *dE, dnrE*.

We first focused on the role of the various regulators for the transcription of the denitrification gene cluster consisting of the *nap* operon, encoding the periplasmic nitrate reductase (EC 1.7.99.4), the *nir* operon, encoding the nitrite reductase (EC 1.7.2.1), the *nor* operon encoding the nitric oxide reductase (EC 1.7.2.5), and the *nos* operon coding for the nitrous oxide reductase protein complex (EC 1.7.2.4; Figure 5). The different transcriptional units were deduced from the RNA sequencing data (Figure 5) and visualized using TRAV (Dietrich et al., 2014). The RNA sequencing experiments revealed a 10- to 100-fold increase in transcription for the denitrification genes under anaerobic conditions. Involved promoter regions were deduced. These results were combined with a bioinformatical transcription factor binding site prediction using the Virtual Footprint tool of the PRODORIC database (Münch et al., 2005). The expression of the *napDAGHBC* operon was found induced by FnrL and significantly repressed by DnrD, DnrE, and DnrF under anaerobic conditions (Figure 5A). This indicated an antagonistic function of FnrL and Dnr at the *napD* promoter. A possible regulatory binding site was found 96.5 bp upstream of the translational start site of *napD* within the *napF* transcript (Figure 5B). The palindromic sequence 5′-**TTGA**T-N_4_-A**TCAA**-3′ exhibit a high degree of similarity to the Anr/Dnr binding site of *P. aeruginosa* (bold letters; Winteler and Haas, 1996; Rompf et al., 1998; Trunk et al., 2010). In contrast, FnrL/Dnr-independent transcription of the *napF* gene was observed. The gene *napF* encodes a cytoplasmic Fe-S protein that was also found in *E. coli* and *R. sphaeroides* 2.4.1. A strong cooperative anaerobic induction by both FnrL and DnrD was observed for *apbE1, cycA1*, and *hemA3* genes as well for the *nirSECFDGHJN, norCBQDE*, and *nosRZDFYLX* operons (Figure 5). *In silico* promoter analyses revealed the palindromic sequence 5′-**TT**A**A**T-N_4_-CAG**AA**-3′ 43.5 bp upstream of the transcriptional start site of the *apbE1* gene as potential binding site for FnrL and/or DnrD (Figure 5). A coupled transcription with *cycA1* encoding a cytochrome C2 was not observed. Despite the anaerobic transcriptional activation of *cycA1* by FnrL and DnrD a potential regulator binding site was not found (Figure 5). Next, the *nirSECFDGHJN* operon and the divergently transcribed *nosR2* gene, encoding flavin-containing nitrous oxide reductase maturation factor (Zhang et al., 2017), were found anaerobically induced by FnrL and DnrD. Within their shared upstream regions one potential binding site 5′-**TT**A**AC**-N_4_-**GTCAA**-3′ was identified. The sequence motive is located 39.5 bp upstream of the transcriptional start site of *nirS* and 41.5 bp upstream of the transcriptional start site of *nosR2*, both almost optimal positions for Fnr-dependent promoters (Tielen et al., 2012; Figure 5). A coupled transcription of *nosR2* and the following *norCBQDE* was indicated by the RNA sequencing data. Nevertheless, an additional Fnr/Dnr binding motif 5′-**TTGAC**-N_4_-**GT**T**AA**-3′ was found 67.5 bp upstream of the *norC* translational start site, allowing for *nosR2*-independent NorCB formation. Another Fnr binding site 5′-**TT**A**AC**-N_4_-**GTCAA**-3′ was found located 41.5 bp upstream of the transcriptional start site of the *hemA3* gene, encoding the heme biosynthetic enzyme 5-aminolevulinic acid synthase and 39.5 bp upstream of the transcriptional start site of the *dnrE* gene, which is divergently transcribed to *hemA3* (Figure 5). RNA sequencing data indicate a coupled transcription of *dnrE*, the open reading frame Dshi_3192 and the *nosRZDFYLX* operon. The transcription of *hemA3, dnrE*, Dshi_3192, and the *nosRZDFYLX* operon was found induced under anaerobic conditions by FnrL and partly by DnrD. Interestingly, *dnrD* gene expression was constitutive and not dependent on oxygen tension. Thus, we identified the first part of aerobic-anaerobic regulatory cascade, in which constitutively produced FnrL and DnrD induce *dnrE* transcription upon anaerobiosis and denitrification. Similarly, *dnrF* transcription is controlled by FnrL and Dnr. As outlined above DnrE and DnrF are repressing *nap* operon expression. Moreover, a significant solely DnrF-dependent transcriptional repression of the *nosZDFYLX* operon was observed (summarized in **Figures 8**, **10**). Since only DnrF mediates repression of the *nos* operon via this palindromic sequence, one could predict this regulation as DnrF specific. Noteworthy is the fact that those two bidirectional Fnr/Dnr binding sequences between *nirS* and *nosR2* as well as between *hemA3* and *dnrE* are controlling the entire denitrification process. These palindromic sequences exhibit an overall identity of 85% (**TTGAC**(G/T)TT(T/G)**GT**T**AA**) with a single inversion of the central four nucleotides. Due to the promoter specificity of FnrL and DnrD an overlapping bidirectional active binding motive was suggested.

### Expression of the *nirS* and *nosR2* promoter is under the control of one central bidirectional functional FnrL/Dnr binding site

**Figure 6:**
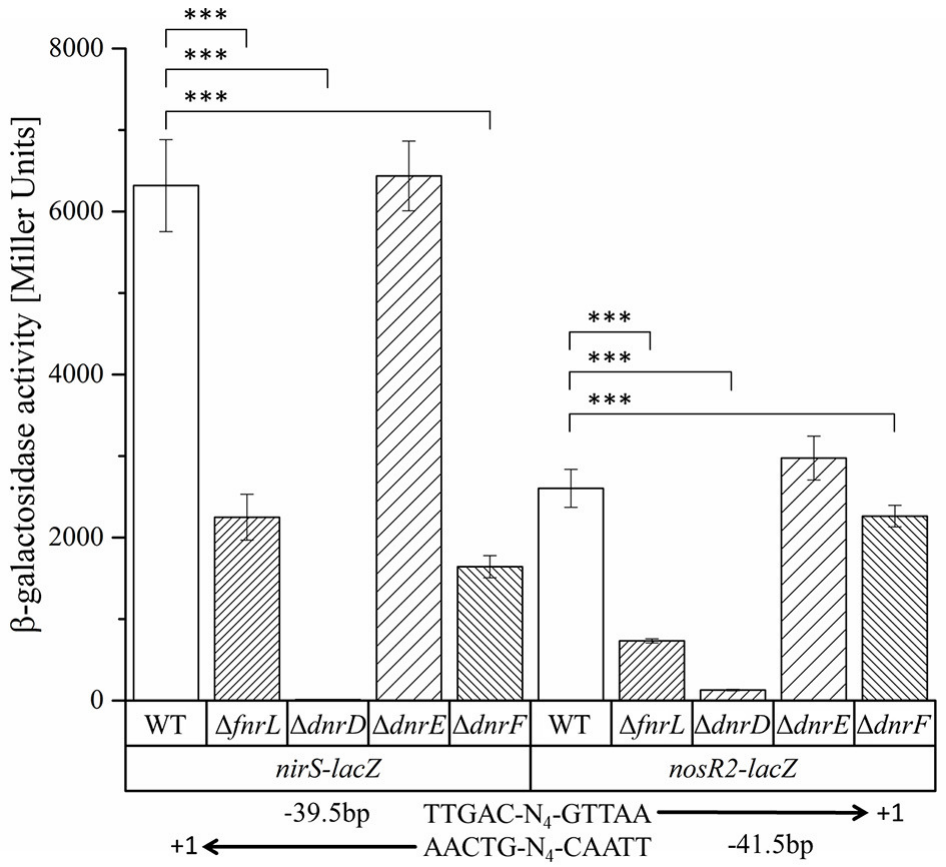
**Functional investigation of the ***nirS***–***nosR2*** intergenic promoter region using ***nirS-lacZ*** and ***nosR2-lacZ*** reporter gene fusions**. *D. shibae* DFL12^T^ wild type (WT), DS001(Δ*fnrL*) (Δ*fnrL*), DS002(Δ*dnrD*) (Δ*dnrD*), DS003(Δ*dnrE*) (Δ*dnrE*), and DS004(Δ*dnrF*) (Δ*dnrF*) mutant strains carrying the *nirS-lacZ* and *nosR2-lacZ* reporter gene fusions were grown under oxygen-limited conditions, and β-galactosidase activity was measured. Error bars represent the observed standard deviation. *P*-values were determined using ANOVA and the Tukey-test (Tukey, 1949). The symbol ^***^ indicate *P*-values smaller or equal to 0. The palindromic sequence of the Fnr/Dnr binding site and its position with respect to the transcriptional start site of *nirS* and *nosR2* are given.

**Figure 7:**
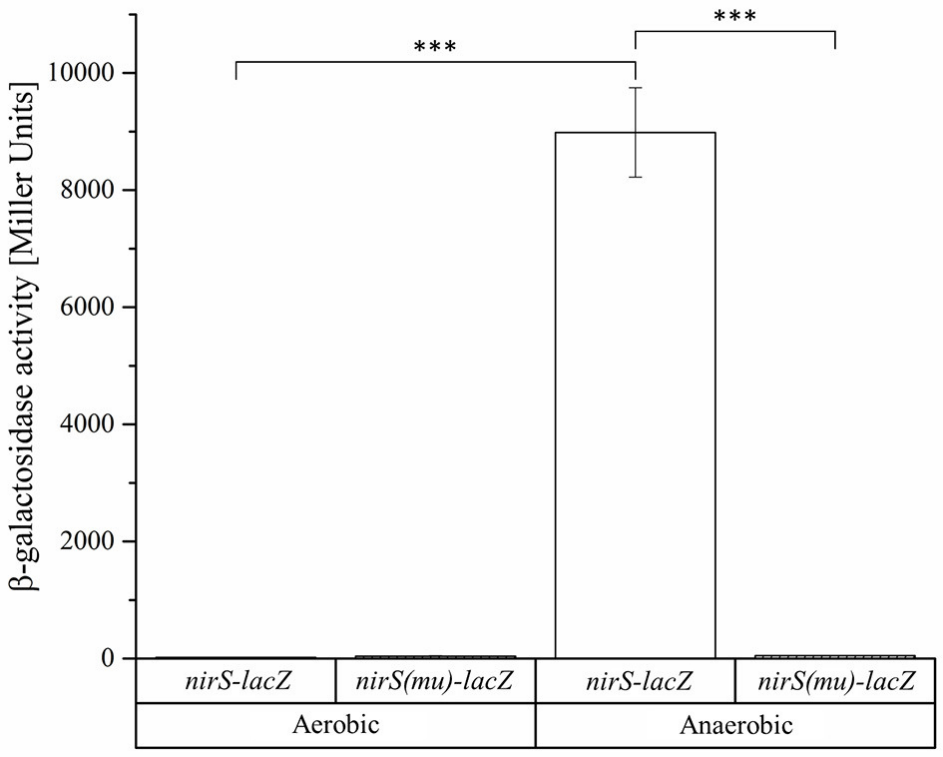
**Functional importance of the Fnr/Dnr binding site in the intergenic region of ***nirS*** and ***nosR2*****. *D. shibae* wild type strains DFL12^T^ carrying the *nirS-lacZ* and *nirS(mu)-lacZ* reporter gene fusions were grown under aerobic and anaerobic growth conditions and β-galactosidase activities were measured in three independent replicates. The *nirS-lacZ* reporter gene fusion is carrying the palindromic sequence 5′-TTAAC-N_4_-GTCAA-3′ of the Fnr/Dnr binding site. The binding sequence was mutated to 5′-**GC**AAC-N_4_-GTC**GC**-3′ in the *nirS(mu)-lacZ* reporter gene fusion. Error bars represent the observed standard deviation. *P*-values were determined using ANOVA and the Tukey-test (Tukey, 1949). The symbol ^***^ indicate *P*-values smaller or equal to 0.

Obviously, the bidirectional employed promoter regions and corresponding Fnr/Dnr binding sites between *nirS* and *nosR2* and between *hemA3* and *dnrE* were predicted to control the onset of denitrification in *D. shibae*. To study the role of the centrally located potential bidirectional binding site between the divergently *nirS* and *nosR2* genes, we created appropriate promoter-*lacZ* reporter gene fusions. We choose a 118 bp DNA fragment spanning the promoter sequences between the transcriptional start site of *nirS* and *nosR2*. The DNA sequence was cloned in both directions upstream of the *lacZ* gene resulting in *nirS*-*lacZ* and *nosR2*-*lacZ* reporter gene fusions (Figure 6). The reporter gene fusions were transformed into the *D. shibae* wild type strain and all four regulator mutant strains: DS001(Δ*fnrL*), DS002(Δ*dnrD*), DS003(Δ*dnrE*), DS004(Δ*dnrF*). The resulting strains were grown under anaerobic conditions in the presence of 25 mM nitrate and ß-galactosidase activities were determined in the mid-exponential growth phase. In the wild type strain *nirS*-*lacZ* expression resulted in 6,318 ± 564 Miller Units. In the DS001(Δ*fnrL*) mutant strain still 2,250 ± 282 Miller Units were determined while in DS002(Δ*dnrD*) *nirS*-*lacZ* expression was totally abolished with 11 ± 0.45 Miller Units. In the DS003(Δ*dnrE*) mutant strain *nirS*-*lacZ* expression was comparable to wild type levels. In the DS004(Δ*dnrF*) mutant strain 1,643 ± 428 Miller Units were measured. Overall expression of *nosR2-lacZ* was lower compared to *nirS*-*lacZ*. In the wild type strain 2,603 ± 233 Miller Units were measured. But again, expression was fund reduced in DS001(Δ*fnrL*) with 732 ± 26 Miller units and almost absent in DS002(Δ*dnrD*). In contrast, wild type levels were found in DS003(Δ*dnrE*) and DS004(Δ*dnrF*). These results identified DnrD as the main regulator of *nirS-lacZ* and *nosR2-lacZ* expression under denitrifying growth condition and FnrL as an additional co-regulator. To confirm the functional role of the potential Fnr/Dnr binding site within the *nirS* promoter, we mutated the terminal nucleotides of the palindrome TT to GC and AA to GC resulting in the mutant sequence 5′-GCAAC-N_4_-GTCGC-3′. Expression of the mutated *nirS(mu)*-*lacZ* reporter gene fusion was studied in the wild type strain under aerobic and anaerobic growth conditions. Under aerobic conditions neither *nirS*-*lacZ* nor *nirS(mu)*-*lacZ* expression were found, confirming strict anaerobic expression (Figure 7). Under anaerobic conditions *nirS*-*lacZ* expression was induced as expected for the wild type promoter, but no expression was found for *nirS(mu)*-*lacZ*. This indicated the importance of the Fnr/Dnr binding site and especially the relevance of the highly conserved TT and AA sequences for regulator binding.

### Genes encoding the electron transport chain are affected by FnrL and Dnr regulators in *D. shibae*

**Figure 8:**
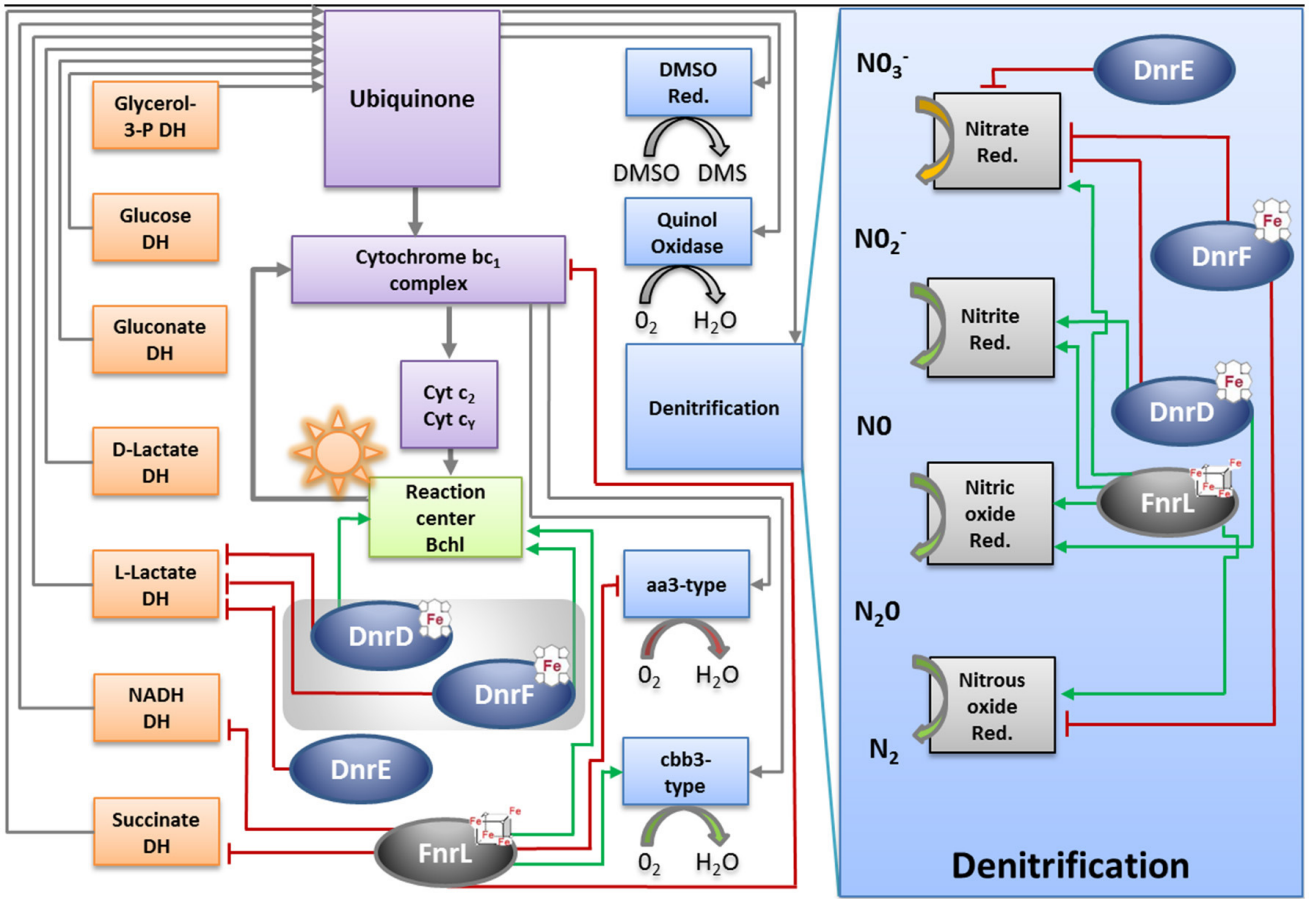
**Model for the regulation of the energy metabolism of ***D. shibae*** by FnrL, Dnr D, DnrE and DnrF during the aerobic to anaerobic transition**. DH, dehydrogenase; Cyt, cytochrome; bchl, bacteriochlorophyll; Q_d_and Q_b_ oxidized and reduced form of reaction center quinone, respectively; Cox, cytochrome oxidase; DMSO, dimethyl sulfoxide; Red, reductase. Figure was adapted from Wagner-Döbler et al. (2010).

In addition to the denitrification genes, genes encoding components of the *D. shibae* electron transport chain were found differentially expressed under anaerobic conditions dependent on FnrL and DnrD (Table S3; Figure 8). The overall composition of the electron transport chain was deduced from the genome of *D. shibae* (Figure 8; Wagner-Döbler et al., 2010). As described previously, the acquisition of electrons occurred by different primary dehydrogenases (Laass et al., 2014). In other bacteria aerobic and anaerobic electron transport chains often employ different electron donor systems. An upregulation of the *nuo*-operon (Dshi_1307-1326; Dshi_1327-1330) encoding an NADH dehydrogenase I (EC 1.6.99.5), Dshi_1390 encoding an alternative NADH dehydrogenase (EC 1.6.5.3 and EC 1.6.99.3) and the *sdh*-operon (Dshi_2861-2867) encoding the succinate dehydrogenase (EC 1.3.99.1) was observed under conditions of anaerobiosis (Figure 8). Interestingly, an FnrL-mediated anaerobic repression of the NADH dehydrogenase (EC 1.6.99.5, EC 1.6.5.3, and EC 1.6.99.3) and the succinate dehydrogenases (EC1.3.99.1) was found. Moreover, the repression of the L-lactate dehydrogenase (*lldD2*; EC 1.1.2.3) was found to be mediated by all three Dnr regulators (Figure 8). Genes for other known primary dehydrogenases remain unaffected by the Fnr and Dnr regulators. An FnrL-dependent repression of the genes encoding an electron transferring flavoprotein (*eftA/B*) was detected. An anaerobic activation of terpenoid backbone synthesis genes (*ispA, crtE, bchP*) was observed which might result in an increase of ubiquinone biosynthesis.

Significant FnrL-dependent repression of transcription of *petC/B* genes encoding the cytochrome bc_1_ complex (EC 1.10.2.2) was observed. Cytochrome bc_1_ channels electrons toward the photosynthesis machinery and two types of cytochrome oxidases using a cytochrome c pool (Figure 8; Tables S1, S3). A considerable FnrL-dependent anaerobic activation of bacteriochlorophyll and carotenoid biosynthesis was noticed. Genes encoding the chlorophyllide a reductase (*bchCXYZ*), the light- independent protochlorophyllide reductase (*bchNB*) and the photosynthetic reaction center (*pufQBALMC*) were found activated by FnrL. Due to a compact genomic organization of photoactive pigments synthesis machinery similar regulatory patterns were observed for phytoene desaturase (*crtBI*), spirillozanthin synthesis (*crtCDE*), and the spheroidene monooxygenase (*crtA*). Furthermore, an FnrL-mediated induced transcription of the *fixNOQPGHIS* operon encoding a high-affinity cbb_3_ type cytochrome oxidase (EC 1.9.3.1) was observed (Figure 8; Table S1, S3). On the other hand a significant anaerobic transcriptional repression of *ctaCBGE* and *ctaD* encoding a low affinity aa_3_-type cytochrome oxidase (EC 1.9.3.1) was found. The final ATP generation by an F_0_F_1_-ATP synthases gene clusters (*atpHAGDC*/*atpIBEXF*; EC 3.6.3.14) was found transcriptionally repressed through FnrL and DnrD (Figure 8; Table S3).

### The essential role of FnrL for the adaptation of *D. shibae* to low oxygen tension

Under anaerobic conditions 477 genes organized within 268 transcriptional units were regulated by FnrL. Taken into account, that only 69 promoters carried a potential Fnr binding site, we assume that the majority of the other genes is regulated in an indirect manner. Thus, the FnrL-specific regulon comprises 69 transcriptional units (Table S2). We performed cluster enrichment analysis of orthologous groups (Blanka et al., 2014; Table S4). In addition to the outlined genes involved in denitrification and corresponding electron transport chains, genes encoding various transcriptional regulators were part of the FnrL regulon. Beside the autoregulation of the *fnrL* gene, an anaerobic activation of the *dnrE* and *dnrF* gene was observed. Moreover, the gene encoding the benzyl coenzyme A (CoA) reduction regulator (*bedM*/*rrf2*) and for quorum sensing regulators (*traR*/*dksA*) were found activated by FnrL. Repression of genes for an ABC transport systems regulator (*rbsB*) and two *luxR* family regulators was observed. In addition, various biosynthetic pathways were FnrL controlled. Genes encoding enzymes of the tetrapyrrole biosynthesis were found activated by FnrL. These include 5-amionlaevulinic acid synthase HemA (Dshi_1182/Dshi_2190) and the coproporphyrinogen III dehydrogenase HemN (Dshi_0541/Dshi_0659), the light-independent protochlorophyllide reductase BchFNBH (Dshi_3533-3536) and chlorophyllide reductase BchXYZF (Dshi_3517-3519/Dshi_3533). Obviously, FnrL is also controlling the restructuring of the cell envelope in response to oxygen limitation. Beside the activation of a gene encoding a putative membrane protein (Dshi_1454) the highest log2-fold change was found for transcription of a gene encoding the outer membrane protein (*ompW*; Bouchal et al., 2010). On the other hand the genes for a part of the protein translocation channel (*secE*), an ABC transporter of unknown function (Dshi_1404) and two metallic cation/iron-siderophore transporters (*znuA*/*sitA*) were found repressed. In addition, the gene of an iron-sulfur cluster assembly accessory protein (Dshi_1730) was found repressed by FnrL. Finally, genes of the cellular stress response are part of the Fnr regulon. For example, genes for universal stress proteins Usp (Dshi_1338/Dshi_2213/Dshi_2686) and genes encoding a cytochrome-c peroxidase (*ccpA*; EC 1.11.1.5) were found highly activated by direct FnrL interaction.

### The role of the three different Dnrs of *D. shibae* for the aerobic-anaerobic transition

**Figure 9:**
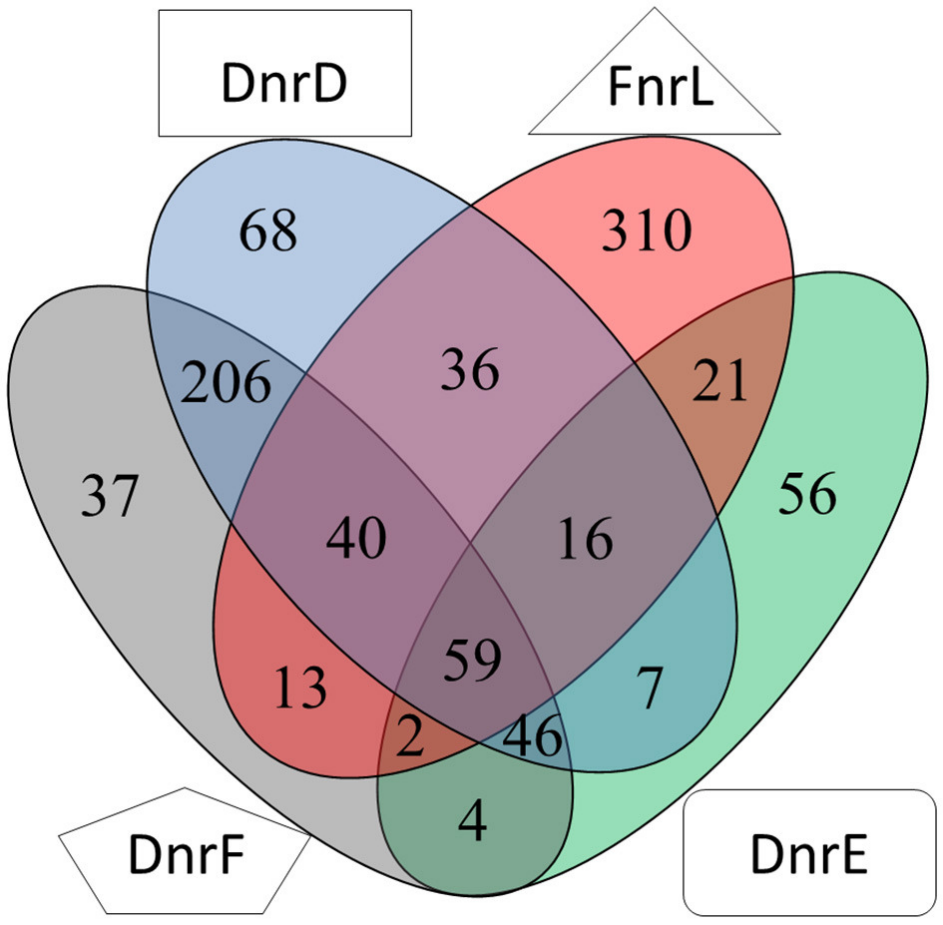
**Venn diagram of the overlapping regulons of Fnr, DnrD, DnrE and DnrF derived from transcriptional profiling experiments**. Numbers for differentially expressed genes regulated by indicated regulators determined by transcriptome analyses using mutants of the corresponding regulator genes compared for aerobic vs. anaerobic growth are given.

Based on the transcriptomic experiments cluster enrichment analyses of orthologous groups were performed (Blanka et al., 2014; Table S4). The determined regulons were merged in a Venn diagram (Figure 9). Especially the DnrD regulator was assumed to be the major player for the regulation of the denitrification processes and the corresponding electron transport chain. We found a set of 68 genes, which were regulated exclusively by DnrD under anaerobic conditions. However, cluster enrichment analyses revealed no orthologous group of genes that was regulated exclusively by DnrD. Nevertheless, a significant activation was observed for detoxification systems like a sulfite export system (Dshi_0205) and a di-haem cytochrome c peroxidase (Dshi_2749). Moreover, we were able to identify 10 potential regulator binding sites for DnrD in front of DnrD regulated genes. A palindromic sequence was found centered 42.5 bp upstream of the transcriptional start of a gene encoding a mineral and organic ion transporter (Dshi_1384). On the other hand pilus assembly genes (Dshi_1130) were found repressed by DnrD (Table S2). A huge overlap of 53% was found for the DnrD and DnrF regulon. Pathways like the carotenoid biosynthesis, the metabolism of terpenoids and polyketides, the degradation of aromatic compounds, cell motility, sulfur metabolism, oxidative phosphorylation, and methane metabolism were found regulated by DnrD and DnrF (Table S2). Furthermore, we found genes for porine biosynthesis (*ompR/*Dshi_0212), environmental information processing (*kspTE*) and homologs recombination (*recA*) differentially expressed in DnrD and DnrF depleted strains. Due to the observed cascade regulation, in which DnrD induces the *dnrF* gene, it was not possible to distinguish between direct DnrD activation and indirect DnrD activity via *dnrF* activation and subsequent DnrF activity. A surprisingly small DnrF specific regulon was deduced with 37 genes. Processes like the biosynthesis of secondary metabolites, signal transduction, iron homeostasis (*hmuS, iscR*, Dshi_0882) and the formation of the quinoprotein glucose dehydrogenase (*gcd*; EC: 1.1.5.2) were found controlled by DnrF. Potential Fnr/Dnr regulator binding sites and significant transcriptional activation were found for eight promoters. Repression of fermentation processes via the downregulation of the genes of an alcohol dehydrogenase class III (*adhC*/Dshi_0473) and changes in the osmotic adaptation via the repression of L-carnitine dehydratase formation (Dshi_3269) were observed (Table S2). The genes of DnrD and DnrE were found in close neighborhood on the genome and exhibit a high amino acid sequence homology. Nevertheless, only a limited overlap of regulons of both regulators was observed (Figure 9). However, the enrichment analysis of DnrE revealed a major impact on translation, lipid metabolism and biosynthesis of amino acids. Interestingly, 73.58% of the specific DnrE regulated genes were located on the various plasmids of *D. shibae*. Genes for cytochromes b_561_ (Dshi_4169), heme uptake (Dshi_4225) and proton/sodium antiporter synthesis (*phaACEFG*) were found exclusively altered in a *dnrE* mutant strain. Moreover, a significant correlation of the observed gene expression and found regulator binding site in the upstream region of the corresponding genes was observed. These data suggest that *D. shibae* possesses a specific regulator for extrachromosomal genes which are essential for the adaptation process to various oxygen tensions (Ebert et al., 2013).

## Discussion

Multiple alternative respiratory and fermentative systems enable bacteria to grown in the absence of oxygen. A broad variety of electron donating primary dehydrogenases, various electron transferring quinones and many different terminal oxidases using alternative electron acceptor including nitrate, sulfate, fumarate, and various metals have been observed. Similarly, bacterial aerobic respiration is using multiple electron-donating and excepting enzyme systems. Depending on the ecological niche inhabited by the bacterium different combinations of these electron transport chains are employed. Often bacteria are capable of aerobic and anaerobic growth. Consequently, a shift from aerobic to anaerobic growth requires a fined-tuned restructuring of the involved electron transport chains with their multiple protein and cofactor components. Parameters like oxygen tension, the presence of nitrate, NO or the electron flux in the membrane provide the bacterium with necessary information to induce the required regulatory process. Species-specific combinations of multiple regulatory proteins are used by the different bacteria to induce a tailor-made regulatory response (see Section Introduction for details).

**Figure 10:**
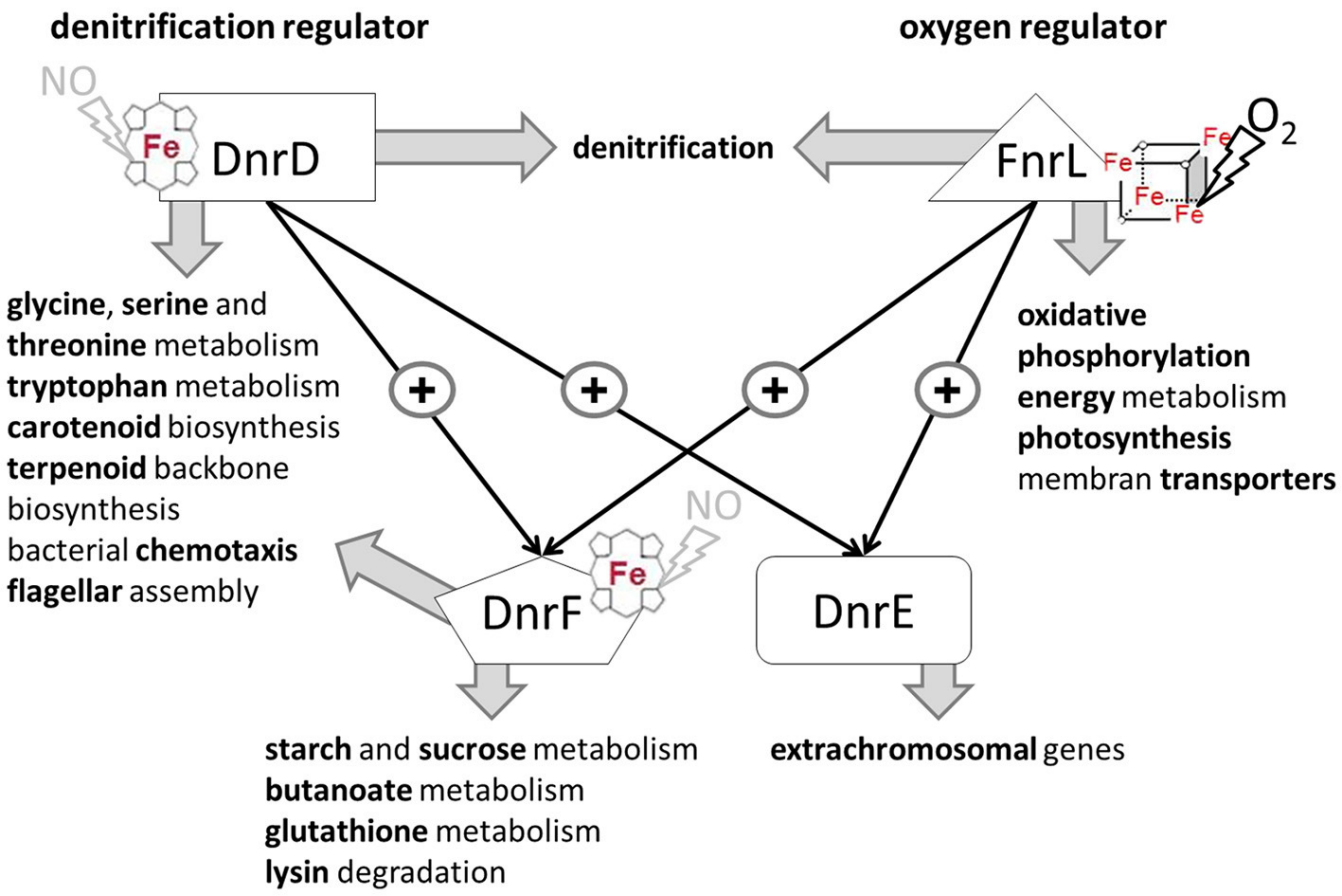
**Cascade type of regulatory network for the control of the aerobic-anaerobic transition by the Crp/Fnr like regulators in ***D. shibae*** DFL12^**T**^**. FnrL sensing oxygen via a bound oxygen-labile Fe-S cluster is the main oxygen regulator. DnrD most likely sensing NO via a bound heme cofactor is the main regulator for denitrification. the master regulators of the aerobic-anaerobic transition. They are inducing/repressing multiple transcriptional units of indicated processes. Moreover, expression of *dnrE* and *dnrF*, which in turn are controlling their own regulons, are modulating the activity of FnrL and DnrD. Interestingly, DnrE is mainly controlling plasmid-encoded genes.

Here we describe for the first time a regulatory network solely composed of four Crp/Fnr-family regulators. It allows the marine bacterium *D. shibae* the transition from aerobic to anaerobic growth. Like in many other bacteria oxygen tension is detected via an oxygen-labile Fe-S cluster attached to an Fnr-type regulator, here FnrL (Härtig and Jahn, 2012; Tielen et al., 2012). Similar to the regulatory network of *P. aeruginosa* and *P. stutzeri* a second Crp/Fnr family regulator termed Dnr, here DnrD, detecting NO via a bound heme cofactors, is required for the full induction of the alternative respiratory system of denitrification (Zumft, 1997; Schreiber et al., 2007; Trunk et al., 2010). However, the nitrate responsive NarX/L system, present in *Pseudomonas*, is missing in *D. shibae*. No genes for other known systems of nitrate detection or membrane associated electron flux measurements, like ArcAB, were detected. The periplasmic nitrate reductase of the Nap type convert available nitrate to nitrite. Chemical conversion of nitrite generates NO, the signal for DnrD. In the absence of oxygen this regulator in combination with FnrL induces the whole denitrification pathway (Figure 8). FnrL controls the formation or repression of other terminal oxidases. No dedicated regulator for the repression of genes of the aerobic energy metabolism was observed. This task was achieved by the whole network. For this purpose FnrL and DnrD are inducing the production of two additional Dnrs, namely DnrE and DnrF (Figure 10). This is also the pre-requisite for the fine-tuned adaption of the set of primary dehydrogenases, the coordination with the anoxygenic photosynthesis (Figure 8) and the usual stress response (Usps, cold shock, glutathione) accompanying major physiological changes. Major overlapping regulons were detected for the four Crp/Fnr family regulators of *D. shibae* (Figure 9) allowing for a tightly and cooperatively controlled gene expression. DnrF induces a Na^+^/H^+^ antiporter system. In agreement, a transposon mutagenesis approach with *D. shibae* identified various genes of Na^+^ gradient formation and utilization as essential for anaerobic growth (Ebert et al., 2013). This provides further evidence for a role of membrane localized Na^+^ gradient in the adaptation to anaerobic growth. The transposon mutagenesis approach also identified multiple essential genes of *D. shibae* for anaerobic growth encoded by the five plasmids of the bacterium (Ebert et al., 2013). In agreement, the activity of one of the Dnr regulators, DnrE, is dedicated to plasmid gene control. Obviously, due to the slower anaerobic growth protein biosynthesis, amino acid biosynthesis, and ATP generation are slowed down (Laass et al., 2014). Corresponding gene regulatory adaptations, with decreased ribosomal protein and ATPase formation have been observed. However, only parts of the observed gene regulatory scenario can be attributed to the direct activity of the four Crp/Fnr regulators, most likely other processes including stringent response might account for the observed adaptation. Overall, a novel type of gene regulatory network composed of one Fnr and three Dnr proteins is tightly controlling the aerobic to anaerobic transition of the marine bacterium *D. shibae*.

## Author contributions

ME, EH, and DJ: Substantial contributions to the conception and design of the work, data collection, data analysis, and interpretation, drafting the article critical revision to the article, final approval to the version to be published. SL, DE, LR, and AT: Contributions to conception and design of the work, data collection, data analysis, and interpretation. RD: Contribution to design of the work, data analysis, and interpretation.

## Funding

This work was supported by the DFG in Transregio-SFB TRR51.

### Conflict of interest statement

The authors declare that the research was conducted in the absence of any commercial or financial relationships that could be construed as a potential conflict of interest.
